# Fluorophore-Assisted Click Chemistry through Copper(I) Complexation

**DOI:** 10.3390/biom10040619

**Published:** 2020-04-16

**Authors:** Victor Flon, Magalie Bénard, Damien Schapman, Ludovic Galas, Pierre-Yves Renard, Cyrille Sabot

**Affiliations:** 1 Normandie Univ, CNRS, UNIROUEN, INSA Rouen, COBRA (UMR 6014), 76000 Rouen, France; victor.flon@insa-rouen.fr (V.F.); pierre-yves.renard@univ-rouen.fr (P.-Y.R.); 2 Normandie Univ, UNIROUEN, INSERM, PRIMACEN, 76000 Rouen, France; magalie.benard@univ-rouen.fr (M.B.); damien.schapman@univ-rouen.fr (D.S.); ludovic.galas@univ-rouen.fr (L.G.)

**Keywords:** click chemistry, CuAAC, chelating fluorophore, chelating azide, fluorescent labeling, azaphthalimide, Kondrat’eva ligation

## Abstract

The copper-catalyzed alkyne-azide cycloaddition (CuAAC) is one of the most powerful chemical strategies for selective fluorescent labeling of biomolecules in in vitro or biological systems. In order to accelerate the ligation process and ensure efficient formation of conjugates under diluted conditions, external copper(I) ligands or sophisticated copper(I)-chelating azides are used. This latter strategy, however, increases the bulkiness of the triazole linkage, thus perturbing the biological function or dynamic behavior of the conjugates. In a proof-of-concept study, we investigated the use of an extremely compact fluorophore-based copper(I) chelating azide in order to accelerate the CuAAC with concomitant fluorescence labeling; in our strategy, the fluorophore is able to complex copper(I) species while retaining its photophysical properties. It is believed that this unprecedented approach which was applied for the labeling of a short peptide molecule and the fluorescent labeling of live cells, could be extended to other families of nitrogen-based fluorophores in order to tune both the reaction rate and photophysical characteristics.

## 1. Introduction

Copper-catalyzed alkyne-azide cycloaddition (CuAAC), also known as click chemistry [1], has attracted tremendous interest in recent years for the site-specific, in vitro modification of biomolecules, such as proteins, glycans, lipids, or nucleic acids, and for the bioorthogonal fluorescent labeling of cell extracts or living systems [2,3,4]. Prominent applications in this latter area include, among others, the understanding of diverse biological processes [5,6], the development of detection tools [7], and real-time live-cell imaging and therapy [8,9,10,11,12]. In contrast to other successful bioorthogonal chemistry, such as strain promoted alkyne azide cycloaddition (SPAAC) [13] and tetrazine-based inverse electron demand Diels−Alder ligation (IEDDA) [14,15], CuAAC ligation has been widely adopted due to the smallness and inertness of the alkyne and azide handles that can be incorporated into biomolecules by using the genetic code expansion, or the cellular metabolic machinery [16], and the fact that small triazole adducts also impose a minimal perturbation of resulting conjugates.

**Figure 1:**
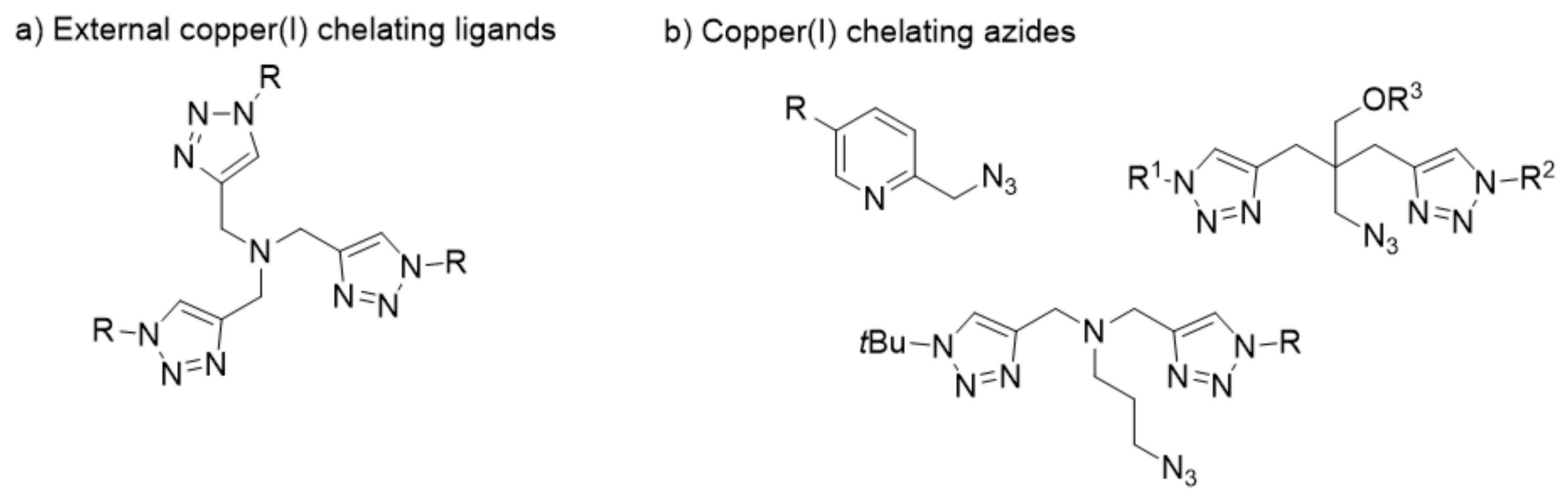
Strategies of copper ligands.

Reaction of alkynes with azides generally involves the in situ formation of copper(I) catalyst from a copper(II) source (e.g., CuSO_4_) in the presence of reducing agent, sodium ascorbate. Although coordinating ligands are not strictly required for CuAAC [5], their combined use has shown to significantly accelerate the alkyne-azide cycloaddition, which thus ensures efficient bioconjugation under diluted conditions imposed by biological systems, while preventing both the deactivation of the copper(I) catalyst by biomolecules and copper-mediated oxidative damage. Tris(triazolylmethyl)amine derivatives, such as TBTA or its water-soluble analogues THPTA, and BTTAA, constitute an important family of such accelerating ligands (Figure 1a) [17,18,19].

However, ligands, copper sources, and a fluorescent bioorthogonal handle are generally required in excess amounts relative to the chemical reporter group for the bioconjugate reaction to proceed accordingly with high yields [20]. These ligands are mostly used for cell-surface labeling, or require covalent attachment to cell-penetrating peptides to facilitate their cellular uptake [21]. To circumvent these limitations, another strategy consists of using azides equipped with a chelating moiety which are capable of complexing the copper(I) species and thus accelerate the reaction in the absence of external ligands by both facilitating the formation of the metallacycle intermediate and increasing the electrophilicity of the azide function [22,23,24]. Highly performant azide chelating systems were designed in this context (Figure 1b) [25,26]. Nevertheless, these chelating azides are constituted of polycyclic ligands with aromatic characters, and when linked to bulky and rigid fluorophores, water-solubilizing groups are required to counterbalance their overall hydrophobicity. As a consequence, subsequent biological, physico-chemical properties of the corresponding conjugates, in particular, for short labeled biomolecules or pharmacophores, may be dramatically altered, which questions the benefits of using small alkyne and azide handles in CuAAC, with respect to other biorthogonal reactions [27].

**Figure 2:**
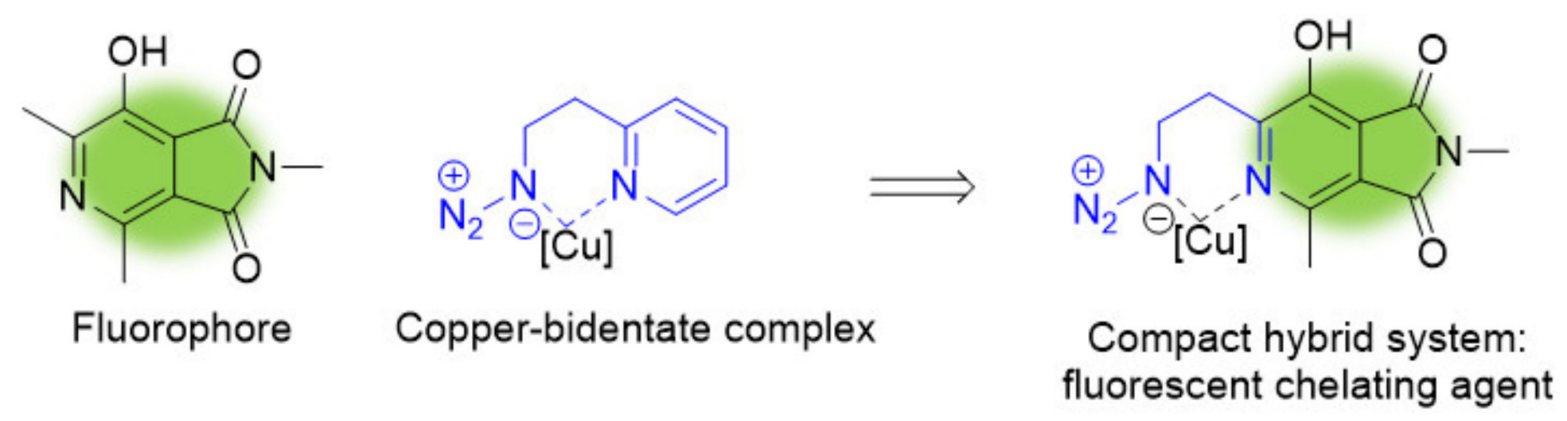
Fluorophore-based cooper(I) ligand for CuAAC-mediated fluorescent labeling.

Following these considerations, in order to perform bioconjugate reactions with alkyne modified biomolecules, we want to report the unprecedented use of an extremely compact, fluorophore-based ligand capable of complexing copper(I) species while retaining its photophysical characteristics useful for bioimaging applications. We previously reported the Kondrat’eva ligation based on a one-pot Diels-Alder/aromatization of 5-alkoxyoazole with maleimide to furnish the corresponding azaphthalimide fluorophore, which has found different applications in chemical biology [28,29]. Interestingly, this small bicyclic azaphthalimide dye displays relatively high excitation and emission wavelengths (ex. 420 nm, em. 520 nm) suitable for live-cell imaging. Herein, we wish to combine both the fluorescent properties of the azaphthalimide dye and its underestimated copper-binding ability to design a readily-available and extremely compact fluorescent copper-chelating azide (Figure 2).

## 2. Materials and Methods

### 2.1. General Information

All chemicals were used as received from commercial sources without further purification. Solvents, unless otherwise stated, were purchased at reagent grade or HPLC grade and used as received, except tetrahydrofuran, which was freshly distilled over sodium prior to use. PBS (pH 7.4, 0.1 M) and aqueous mobile phases for HPLC were prepared with water purified by means of a MilliQ system (purified to 18.2 MΩ cm). All reactions were monitored by thin layer chromatography (TLC) and/or RP-HPLC. TLC were carried out on Merck DC Kieselgel 60 F-254 aluminum sheets. Visualization of spots was performed under a UV lamp at λ = 254 or 365 nm, and/or staining with a KMnO_4_ solution/K_2_CO_3_ + 5% NaOH, and developed with heat. Flash column chromatography purifications were performed manually on silica gel (40–63 µM) under pressurized air flow.

Instruments and Methods. HPLC system A: RP-HPLC analyses were performed with a Thermo Fischer Ultimate 3000 RS instrument (Villebon sur Yvette, France), equipped with a diode array detector (DAD-3000RS). The temperature of the column compartment was fixed at 25 °C. A Thermo Hypersyl GOLD^®^ C18 column (1.9 µm, 2.1 × 50 mm) (Illkirch-Grafenstaden, France) was used with a binary solvent system composed of MeCN and 0.1% aqueous formic acid (aqueous FA, pH 2) as eluents (linear gradient from 5 to 100% MeCN over 10 min) at a flow rate of 0.600 mL/min. System B: Semi-preparative RP-HPLC was performed with an Interchim puriFlash^®^ 4250 instrument (Montluçon, France) equipped with a diode array detector, and a Thermo Hypersyl GOLD^®^ C18 column (5 µm, 30.0 × 250 mm) with MeCN and water as eluents (linear gradient 10%–65% MeCN for 50 min) at a flow rate of 40 mL/min. System C: Semi-preparative RP-HPLC was performed with an Interchim puriFlash^®^ 4250 instrument equipped with a diode array detector, and a Thermo Syncronis^®^ C18 column (5 µm, 21.2 × 250 mm) with MeCN and 0.1% aqueous FA as eluents (linear gradient 5%–95% MeCN for 35 min) at a flow rate of 15 mL/min. System D: Semi-preparative RP-HPLC was performed with an Interchim puriFlash^®^ 4250 instrument equipped with a diode array detector, and a Thermo Hypersyl GOLD^®^ C18 column (5 µm, 20.0 × 250 mm) with MeCN and water as eluents (linear gradient 25–70% MeCN for 25 min) at a flow rate of 40 mL/min. System E: Semi-preparative RP-HPLC was performed with a Thermo Scientific SPECTRASYSTEM liquid chromatography system (P4000) equipped with a UV–Vis 2000 detector, and a Hypersyl GOLD^®^ C18 column (5 µm, 20.0 × 250 mm) with MeCN and 0.1% aqueous FA as eluents (linear gradient 10%–90% MeCN for 30 min) at a flow rate of 10 mL/min.

High resolution mass spectrometry (HRMS) were obtained by using a Waters Micromass LCT Premier XE^®^ (Manchester, United Kingdom) equipped with an orthogonal acceleration time-of-flight (oa-TOF) and an electrospray source in positive mode.

^1^H, ^13^C, and NMR spectra were recorded on Bruker 300 machine operating at ambient temperature. The solvent resonance was used as the internal standard for ^1^H-NMR (chloroform-d_3_ at 7.26 ppm; DMSO-d_6_ at 2.50 ppm; MeOD-d_4_ at 3.31 ppm) and ^13^C-NMR (chloroform-d_3_ at 77.0 ppm; DMSO-d_6_ at 29.8 ppm; MeOD-d_4_ at 49.0 ppm). Chemical shifts (δ) were quoted in parts per million (ppm). Coupling constants (*J*) were quoted in Hertz (Hz). The following abbreviations were used to give the multiplicity of the NMR signals: s: singlet, bs: broad singlet, d: doublet, t: triplet, dd: doublet of doublets.

Fluorescence spectroscopic studies (emission/excitation spectra and time course for kinetics monitoring) were performed on a Varian Cary Eclipse^®^ spectrophotometer (Le Plessis-Robinson, France) using a quartz fluorescence cell (Hellma, Jena, Germany, 104F-QS, 10 × 4 mm, lightpath: 10 mm, chamber volume 1.4 mL) and excitation/emission spectra were recorded at 20 °C. UV-Vis absorption spectra were obtained on a Varian Cary 60 UV–Vis^®^ (Agilent, Les Ulis, France) using a standard cell (10 × 10 mm, chamber volume 3.5 mL) at 20 °C.

Fluorescence quantum yields were measured on a HORIBA Fluorolog 3 spectrophotometer (Longjumeau, France) with a quartz fluorescence cell (Hellma, 104F-QS, lightpath: 10 × 10 mm, chamber volume: 3.5 mL, excitation and emission slit: 2 nm) at 25 °C by a relative method using Lucifer Yellow (*Φ*_F_ = 21% in Water, 430 nm) [30] as a standard. The following equation was used to determine the relative fluorescence quantum yield:

*Φ*_F_ (X) = (A_s_/A_x_) (F_x_/F_s_) (n_x_/n_s_)^2^*Φ*_F_ (S)

where A is the absorbance (in the range of 0.01–0.1 a.u.), F is the area under the emission curve, n is the refractive index of the solvents (at 25 °C) used in measurements, and the subscripts s and x represent standard and unknown, respectively. The following refractive index values were used: 1.337 for PBS 0.1 M pH 7.4.

### 2.2. Chemical Synthesis

Compounds **6** [25], **7** [31], **10** [25], and **14** [32] were prepared as described previously.

*Ethyl 2-(5-ethoxy-2-methyloxazol-4-yl)acetate***2**. To a suspension of P_2_O_5_ (1.85 g, 13 mmol, 3 equiv.), CaO (1.25 g, 22 mmol, 5 equiv.), and Celite^®^ (400 mg) in dry chloroform (40 mL) stirred vigorously at room temperature, was added diethyl *N*-acetylaspartate (1 g, 4.32 mmol) dissolved in dry CHCl_3_ (5 mL). The mixture was brought to reflux for 3 h; then P_2_O_5_, CaO and Celite^®^ were added in the aforementioned amounts, and the reaction mixture was refluxed for further 3 h. The mixture was cooled to 0 °C and a solution of saturated aqueous NaHCO_3_ (100 mL) was added slowly with stirring for 30 min. Water (20 mL) was added and the solution was extracted with DCM (3 × 100 mL). The organic phases were combined and then washed with a solution of saturated aq NaCl (150 mL), dried over MgSO_4_ and filtered through Celite^®^. The solvents were evaporated to dryness and the crude product was purified by chromatography on silica gel in an AcOEt/cyclohexane elution system (1:3 *v*/*v*) to (1:1 *v*/*v*); the desired product was obtained as a yellow oil (620 mg, 2.90 mmol, 67%). The ^1^H RMN analysis is in agreement with the one described in the literature [33]: ^1^H RMN (300 MHz, CDCl_3_) δ = 4.17 (q, *J* = 7.2 Hz, 2 H), 4.14 (q, *J* = 7.2 Hz, 2 H), 3.41 (s, 2 H), 2.34 (s, 3 H), 1.36 (t, *J* = 7.2 Hz, 3 H), 1.28 (t, *J* = 7.2 Hz, 3 H).

*2-(5-Ethoxy-2-methyloxazol-4-yl)ethan-1-ol***3**. To a suspension of LiAlH_4_ (810 mg, 21.30 mmol, 2.5 equiv.) in anhydrous THF (120 mL) cooled at 0 °C, the oxazole ethyl 2-(5-ethoxy-2-methyloxazol-4-yl)acetate **2** (1.8 g, 8.53 mmol) in dry THF (20 mL) was added dropwise. After 15 min at 0 °C, the solution was allowed to warm at room temperature and further stirred for 3 h. Then, the mixture was cooled to 0 °C, and water (5 mL), a solution of aqueous NaOH (5 M, 5 mL), and water (15 mL) were added successively. The mixture was stirred for 15 min; then MgSO_4_ was added. The resulting cake was filtered through Celite^®^, washed with ethyl acetate, and the filtrate was evaporated to dryness. The crude reaction mixture was then purified quickly by chromatography on a short plug of silica gel (AcOEt) and the desired product was obtained as yellow oil (650 mg, 3.80 mmol, 46%). ^1^H RMN (300 MHz, CDCl_3_) δ = 4.09 (q, *J* = 7.2 Hz, 2 H), 3.77 (t, *J* = 6 Hz, 2 H), 2.56 (t, *J* = 6 Hz, 2 H), 2.26 (s, 3 H), 1.30 (t, *J* = 7.2 Hz, 3 H). ^13^C RMN (75MHz, CDCl_3_) δ = 154.1, 152.6, 114.2, 70.5, 61.5, 27.6, 15.0, 14.2. IR (neat, cm^−1^): 3347, 2930, 1671, 1583, 1379, 1264, 1237, 1018, 1049, 603, 473. HRMS (ESI+): calculated for C_8_H_14_NO_3_ [M + H]^+^: 172.0974; found: 172.0972.

*2-(5-Ethoxy-2-methyloxazol-4-yl)ethyl methanesulfonate***4**. To the alcohol 2- (5-ethoxy-2-methyloxazol-4-yl) ethan-1-ol **3** (300 mg, 1.75 mmol) in dry THF (30 mL) at 0 °C, triethylamine (500 μL, 3.68 mmol, 2.1 equiv.) and mesyl chloride (270 μL, 3.50 mmol, 2 equiv.) were successively added dropwise, and the resulting mixture was stirred at room temperature for 3 h. Water (30 mL) was added to the solution, and the mixture was further stirred for 15 min. The aqueous phase was extracted with AcOEt (3 × 20 mL); the organic phases were combined and then washed with a solution of saturated aq NaCl (30 mL), dried over MgSO_4_, and filtered through Celite^®^. The solvent was evaporated to dryness, and the crude product was purified by chromatography on silica gel (AcOEt); the desired product was obtained as a yellow oil (430 mg, 1.72 mmol, 98%). 1H RMN (300 MHz, CDCl_3_) δ = 4.40 (t, *J* = 6.9 Hz, 2 H), 4.13 (q, *J* = 7.2 Hz, 2 H), 2.96 (s, 3 H), 2.80 (t, *J* = 6.9 Hz, 2 H), 2.30 (s, 3 H), 1.35 (t, *J* = 7.2 Hz, 3 H). RMN ^13^C (75 MHz, CDCl_3_) δ = 154.9, 152.8, 111.0, 70.6, 68.1, 37.4, 25.1, 15.0, 14.2. IR (neat, cm^−1^): 2983, 2936, 1673, 1444, 1349, 1267, 1169, 1018, 951, 902, 800, 526. HRMS (ESI+): calculated for C_9_H_15_NO_5_S [M + H]^+^: 250.0749; found: 250.0753.

*4-(2-Azidoethyl)-5-ethoxy-2-methyloxazole***5**. To a solution of oxazole 2- (5-ethoxy-2-methyloxazol-4-yl) ethyl methanesulfonate **4** (400 mg, 1.60 mmol) in dry DMF (8 mL), sodium azide (620 mg, 9.60 mmol, 6 equiv.) was added. After 15 min stirring at room temperature, the solution was heated at 50 °C for 8 h. Then, the mixture was cooled to RT and water (10 mL) was added. The aqueous phase was extracted with AcOEt (2 × 30 mL) and the combined organic phases were washed with a solution of saturated aqueous NaCl (15 mL) and dried over MgSO_4_, filtered through Celite^®^, and then the solvent was evaporated to dryness. The crude reaction mixture was then purified by chromatography on silica gel in a AcOEt/cyclohexane elution system (1:5 *v*/*v* to 1:1 *v*/*v*); the desired product was obtained as yellow oil (240 mg, 1.22 mmol, 76%). ^1^H RMN (300MHz, CDCl_3_) δ = 4.15 (q, *J* = 7.2 Hz, 2 H), 3.5 (t, *J* = 6.9 Hz, 2 H), 2.63 (t, *J* = 6.9 Hz, 2 H), 2.32 (s, 3H), 1.37 (t, *J* = 7.2 Hz, 3 H). ^13^C RMN (75 MHz, CDCl_3_) δ = 154.56, 152.60, 112.74, 70.39, 49.83, 24.75, 15.01, 14.28. IR (neat, cm^−1^): 2979, 2930, 2094 (N3), 1671, 1587, 1379, 1265, 1229, 1171, 1020, 952, 644. HRMS (ESI+): calculated for C_8_H_12_N_4_O_2_ [M + H]^+^: 197.1039; found: 197.1042.

*6-(2-Azidoethyl)-7-hydroxy-2,4-dimethyl-1H-pyrrolo[3,4-c]pyridine-1,3(2H)-dione***1**. *N*-methylmaleimide (47 mg, 0.43 mmol, 1.05 equiv.) was added to a solution of 4-(2-azidoethyl)-5-ethoxy-2-methyloxazole **5** (80 mg, 0.41 mmol) in toluene (1.5 mL). After stirring at room temperature for 15 min and then at 70 °C for 6 h, the mixture was concentrated under reduced pressure. The residue was dissolved in CDCl_3_ (1 mL), and then formic acid (10 μL, 0.265 mmol, 0.65 equiv.) was added. The mixture was allowed to stir at room temperature overnight. The reaction mixture was concentrated under reduced pressure and the resulting residue was purified by semi-preparative RP-HPLC according to system B to afford the desired product (43 mg, 0.16 mmol, 40%) as a yellow solid. Melting point: 107.3–108.5 °C. ^1^H RMN (300 MHz, MeOD) δ = 3.69 (t, *J* = 6.9 Hz, 2 H), 3.16 (t, *J* = 6.9 Hz, 2 H), 3.06 (s, 3 H), 2.67 (s, 3 H) ppm. ^13^C RMN (75 MHz, MeOD) δ = 169.51, 168.58, 157.58, 147.41, 146.43, 122.67, 122.15, 50.07, 32.95, 23.88, 19.77 ppm. IR (neat, cm^−1^): 3304, 2944, 2100 (N3), 1698, 1433, 1382, 1233, 1004, 756, 519. HRMS (API+): calculated for C_11_H_12_N_5_O_3_ [M + H]^+^: 262.0940; found: 262.0946.

*7-Hydroxy-2,4-dimethyl-6-(2-(4-(2-oxo-2H-chromen-7-yl)-1H-1,2,3-triazol-1-yl)ethyl)-1H-pyrrolo[3,4-c]pyridine-1,3(2H)-dione***9**. To a solution of 6- (2-azidoethyl) -7-hydroxy-2,4-dimethyl-1H-pyrrolo [3,4-c] pyridine-1,3 (2H) –dione **1** (13.55 mg, 0.052 mmol) in a solution of H_2_O/t-BuOH (200 μL, 1:1, *v*/*v*), 7-ethynylcoumarin (8.65 mg, 0.052 mmol, 1 equiv.) [1] in DCM (100 μL), sodium ascorbate (10.3 mg, 0.052 mmol, 1 equiv.), and copper sulfate pentahydrate CuSO_4_·5H_2_O (1.3 mg, 0.052 mmol, 1 equiv.) were successively added. The reaction mixture was stirred at room temperature in the darkness for 4 h (analytical RP-HPLC control, system A), and then the crude reaction mixture was concentrated under reduced pressure. The resulting residue was triturated in Et_2_O and then EtOH. The pellet recovered after centrifugation was purified by preparative RP-HPLC according to system C; the desired product (5 mg, 0.12 mmol, 23%) was obtained as a yellow solid. Melting point: 179.9–181.2 °C. ^1^H RMN (300 MHz, DMSO-d_6_) δ = 8.72 (s, 1 H), 8.04 (d, *J* = 9.6 Hz, 1 H), 7.84 - 7.75 (m, 3 H), 6.46 (d, *J* = 9.6 Hz, 1 H), 4.85 (t, *J* = 6.9 Hz, 2 H), 3.49 (t, 2 H), 2.97 (s, 3 H), 2.59 (s, 3 H) ppm. ^13^C RMN (75 MHz, DMSO-d_6_) δ = 168.00, 166.46, 159.94, 155.49, 154.08, 145.72, 144.73, 143.92, 134.31, 129.12, 123.17, 121.17, 118.15, 115.76, 112.13, 47.49, 32.35, 23.56, 19.50 ppm. IR (neat, cm^−1^): 2924, 1706, 1681, 1621, 1430, 1223, 1000, 845, 754, 616, 590, 524, 456. HRMS (ESI+): calculated for C_22_H_18_N_5_O_5_ [M + H]^+^: 432.1308; found: 432.1302.

7*-(1-Phenethyl-1H-1,2,3-triazol-4-yl)-2H-chromen-2-one* **11**. To a solution of 2-phenylethyl azide **7** (89 mg, 0.52 mmol) in a solution of H_2_O/*t*-BuOH (1.5 mL, 1:1, *v*/*v*), 7-ethynyl coumarin (97 mg, 0.52 mmol, 1 equiv.) in DCM (300 μL), sodium ascorbate (104 mg, 0.52 mmol, 1 equiv.), and copper sulfate pentahydrate CuSO_4_·5H_2_O (13 mg, 0.05 mmol, 0.1 equiv.) were added. The reaction mixture was allowed to stir at room temperature in the darkness for 12 h; then concentrated under reduced pressure. The obtained residue was dissolved in AcOEt (1 mL) and then purified by chromatography on silica gel in an AcOEt/cyclohexane elution system (3:1, *v*/*v*) to (3:0, *v*/*v*). The desired product (110 mg, 3.47 mmol, 66%) was obtained as a yellow solid. Melting point: 180–181.9 °C. ^1^H RMN (300 MHz, DMSO-d_6_) δ = 8.71 (s, 1 H), 8.04 (d, *J* = 9.3 Hz, 1 H), 7.79 (t, *J* = 7.8 Hz, 3 H), 7.25 (t, *J* = 6.5 Hz, 5 H), 6.46 (d, *J* = 9.3 Hz, 1 H), 4.68 (t, *J* = 6.9 Hz, 2 H), 3.23 (t, *J* = 6.9 Hz, 2 H) ppm. ^13^C RMN (75 MHz, DMSO-d_6_) δ = 159.9, 154.1, 144.8, 143.9, 137.5, 134.2, 129.1, 128.7, 128.4, 126.6, 122.8, 121.1, 118.1, 115.8, 112.0, 50.8, 35.5 ppm. IR (neat, cm^−1^): 3121, 2924, 1702, 1620, 1455, 1222, 1151, 939, 838, 754, 696, 614, 494. HRMS (ESI+): calculated for C_19_H_16_N_3_O_2_ [M + H]^+^: 318.1243; found: 318.1244.

*Alkyne-ISWLFVR-OH***12**. The 2-chlorotrityl chloride resin (1.0 g, 1.60 mmoles/g, 1.60 mmoles) was swollen for 20 min in DCM (15 mL) under nitrogen bubbling. A solution of the amino acid Fmoc-Arg(pbf)-OH (1.04 g, 1.60 mmoles, 2 equiv.) and DIPEA (1.64 mL, 9.60 mmoles, 6 equiv.) in DCM (10 mL) was added on the resin, and agitated with nitrogen bubbling for 2.5 h at room temperature. The resulting resin was then treated with MeOH (~5 mL) and agitated under nitrogen bubbling for 15 min. Then, the resin was washed successively by DCM/MeOH/DIPEA (15 mL, 17:2:1, *v*/*v*/*v*, repeated 3 times), DCM (3 × 15 mL), DMF (3 × 15 mL), DCM (3 × 15 mL), and MeOH (3 × 15 mL). The resin was dried under vacuum and the attachment level of the first residue was determined by UV-Vis (55%, 0.88 mmoles) [34]. Fmoc-Arg (pbf)-2-chlorotrityl resin (0.88 mmoles, 1 eq) was swollen for 20 min in DCM (15 mL) under nitrogen bubbling and then washed with DMF (3 × 15 mL). Deprotection of the Fmoc group was performed with piperidine 20% in DMF (10 mL) for 1 min with nitrogen bubbling. The resin was filtered, and then deprotection was again performed for 10 min. After washing with DMF (3 × 15 mL), subsequent Fmoc-amino acids were coupled in excess (2.64 mmol, 3 equiv.) on the deprotected amino-acid bearing resin with HBTU (2.95 equiv.) and DIPEA (6 equiv.) in DMF (~5 mL) for 1 h with nitrogen bubbling at room temperature. The coupling reactions were performed twice with a filtration of the resin between them. The resin was washed with DMF (3 × 15 mL), and a new cycle of deprotection/coupling was performed. The final H-peptide-resin was successively washed with DMF (3 × 15 mL), DCM (3 × 15 mL), and MeOH (3 × 15 mL), and then was dried under vacuum. The coupling of the 4-pentynoic acid (87 mg, 0.88 mmoles, 2 equiv.) was performed on half of the N-term primary amine free ISWLFVR-resin (0.44 mmoles, 1 equiv.) with HBTU (326 mg, 0.86 mmol, 1.95 equiv.) and DIPEA (300 μL, 1.76 mmoles, 4 equiv.) in DMF (~5 mL) for 2 h at room temperature, under nitrogen bubbling. The resulting peptide was cleaved from the resin with a solution of TFA/TIS/H_2_O (~10 mL, 95:2.5:2.5, *v*/*v*/*v*) for 2 h at room temperature. The cleavage solution was evaporated, and the peptide was precipitated with cold diethyl ether followed by washing by centrifugation 3 times with diethyl ether (9000 rpm, 10 min). The resulting residue was solubilized in water and lyophilized before a purification by semi-preparative RP-HPLC according to system D. The desired product was obtained as a white solid (62 mg, 0.06 mmol, 14%). HRMS (ESI+): calculated for C_51_H_74_N_11_O_10_ [M + H]^+^: 1000.5620; found: 1000.5628. HPLC System A (λ = 254 nm): *t*_R_ = 3.66 min.

*Azapthalimide-ISWLFVR-OH***13**. To a solution of the peptide-alkyne **12** in DMF (2 mg, 1.25 mL, 1.6 mM, 1 eq) was added a solution of azaphthalimide-azide **1** in DMF (2.04 mg, 1.25 mL, 3.2 mM, 2 equiv.). The mixture was then diluted with PBS buffer solution (2.50 mL, 0.1 M, pH = 7.4), and a solution of copper sulfate pentahydrate in MilliQ water (1 mg, 2.50 mL, 1.6 mM, 2 equiv.) was added. The reaction mixture was stirred at room temperature for 10 min, and then a solution of sodium ascorbate in water (19.81 mg, 2.50 mL, 40 mM, 50 equiv.) was added. The reaction was monitored by analytical RP-HPLC, system A. The crude reaction mixture was then directly purified (without prior treatment) by preparative RP-HPLC according to system E. The desired product (1.2 mg, 0.95 mmol, 38%) was obtained as a yellow solid. HRMS (ESI+): calculated for C_62_H_85_N_16_O_13_ [M + H]^+^: 1261.6482; found: 1261.6497. HPLC System A (λ = 254 nm): *t*_R_ = 3.63 min.

### 2.3. Live Cells Labeling

The PC12 cell line, derived from a pheochromocytoma of the rat adrenal medulla, was cultured in a humidified incubator at 37 °C in an atmosphere of 5% CO2. Cells were grown in Dulbecco’s modified Eagle’s medium (DMEM) (Thermo Fisher Scientific, Illkirch, France) supplemented with 7% heat-inactivated fetal bovine serum (Sigma–Aldrich, Saint-Quentin Fallavier, France), 7% horse serum (Lonza Bioscience, Walkersville, MD, USA), 2.5% HEPES (4-(2-hydroxyethyl)-1-piperazine ethanesulfonic acid) (Thermo Fisher Scientific), 1% glutamine (Thermo Fisher Scientific), 100 units/mL penicillin, and 100 μg mL^−1^ streptomycin (Thermo Fisher Scientific).

PC12 cells were plated on glass bottom dishes (MatTek Corporation, Ashland, Massachusetts, USA) for staining and confocal microscopy observation (SP8). After medium removal and PBS rinse, cells were incubated or not (“control” and “cocktail alone” conditions) for 30 min at 37 °C in an atmosphere of 5% CO2 with a 50 µM Alkyne 1-(4-pentyn-1-yl)-1H-pyrrole-2,5-dione **14** solution in PBS 0.1 M pH 7.4 (10 mL, containing 0.05% acetonitrile) prepared from a 5 mM alkyne **14** in acetonitrile/milliQ water 5:95 (1 mL stock solution). To a “cocktail” solution with azide **1** (50 mM), copper sulfate pentahydrate (50 mM), and sodium ascorbate (1.25 mM), prepared 45 min before incubation (azide 1 stock solution 10 mM in DMSO/water 10:90 (500 mL), copper sulfate pentahydrate stock solution 20 mM in water (250 mL) was added. After 30 min of incubation at 20 °C, the sodium ascorbate stock solution 0.5 M in water (250 mL) was added, and the mixture was further incubated for 15 min at 20 °C. After 2 washes with PBS, cells were incubated during 30 min at 37 °C in an atmosphere of 5% CO_2_ with the “cocktail” solution implemented with 1 µM of the non-ionic surfactant polyol Pluronic F-127 (Thermo Fisher Scientific). Cells were washed one time with PBS before being visualized under a microscope.

Images (1024 × 1024 pixels) were acquired by using an upright fixed-stage Leica TCS SP8 confocal microscope (Leica Microsystems, Nanterre, France) equipped with diode laser at 405 nm to excite the fluorophores and a conventional scanner at 400Hz. Using a 25× objective (NA 0.95, water immersion) fluorescence emission was detected through a hybrid detector (HyD) in photon counting mode with a specific band from 500 to 550 nm. Mosaic image (square of 5 by 5 images at 1024 × 1024 pixels) was performed to obtain a large view, and a merging was realized after acquisition through module LAS X Navigator. For image acquisitions, focusing on cells was performed in bright-field mode before fluorescence acquisition. For the fluorescence quantification, all values are expressed as fluorescence intensity (A.U.) means of at least 100 cells ± sems. Statistical analysis was performed using the GraphPad Prism 4 software (GraphPad Software Inc., San Diego, CA, USA) and a one-way analysis of variance (ANOVA) with a Tukey–Kramer multiple comparisons tests.

## 3. Results and Discussion

### 3.1. Synthesis of the Fluorophore-Based Chelating Azide

**Scheme 1:**
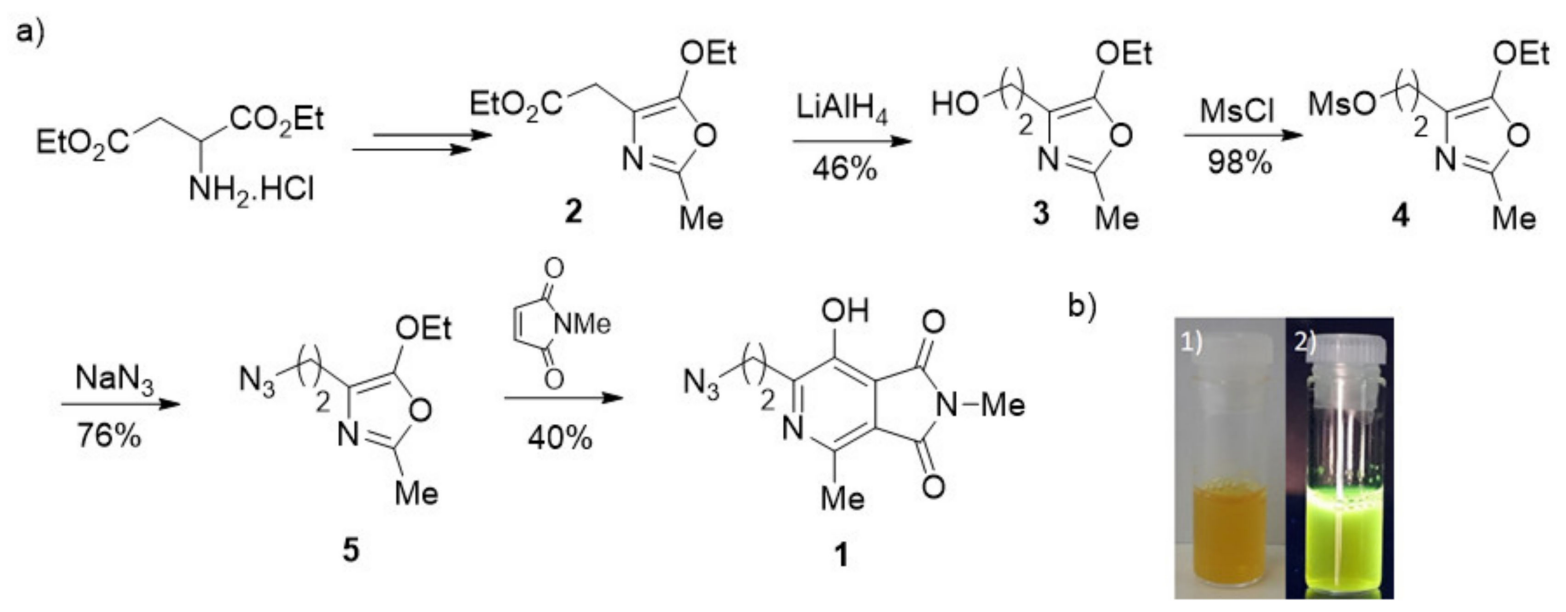
(**a**) Synthetic access to the azaphthalimide **1**. (**b**) Photographs of **1** (1 mg/mL PBS 0.1 M pH 7.4) taken under visible light (1) or under 365 nm light (2).

The fluorescent chelating agent **1** was prepared from commercial L-aspartic acid diethyl ester hydrochloride (Scheme 1). First, the reported oxazole ester **2** obtained in two steps [33], was reduced into the corresponding alcohol derivative **3** in 46% isolated yield. The hydroxyl group was then converted into mesylate intermediate **4**, which was subsequently displaced with azide ion to afford compound **5** in 74% over two steps. Finally, the oxazole **5** underwent a [4+2] cycloaddition/aromatization process with *N*-methylmaleimide to furnish the corresponding azaphthalimide **1** in 40% isolated yield. Importantly, this compound proved to be relatively soluble in PBS pH 7.4 as the only solvent, up to 4 mM (Scheme 1a,b). Interestingly, the Log*S* value of azaphthalimide **1** was found to be −1.58, which is in the range of those calculated for water-soluble tris(triazolylmethyl)amine THPTA and BTTAA (Table S8).

### 3.2. Comparative Study with Chelating and Non-Chelating Azide

Azaphthalimide **1**’s ability to accelerate 1,3-dipolar cycloadditions was compared to a reportedly effective but not fluorescent pyridine-based chelating azide, 2-(2-azidoethyl)pyridine **6**, [25] and a non-chelating derivative, 2-phenylethyl azide **7**. We chose these three azide-based reagents, since in all of them, the azide function is separated from the aromatic ring by a two carbon-atom alkyl chain length in order to form a 6-membered metallocycle system (for chelating azides **1** and **7** only). The fluorogenic 7-ethynyl coumarin **8** whose fluorescence increases upon reaction with azides [25], was chosen as the click partner in order to monitor the click reactions progress by fluorescence spectroscopy. For compounds **6** and **7**, an excitation and emission wavelengths of 320 and 400 nm were selected, corresponding to the absorption and emission of the triazole-substituted coumarin (Figures S2 and S3). With regard to azaphthalimide azide **1**, the coumarin and azaphthalimide scaffold display complementary photophysical properties to constitute a suitable FRET pair with an excitation at the coumarin wavelength (λ_ex_ = 320 nm), and an emission at the azaphthalimide emission wavelength (λ_em_ = 550 nm, Figure S1). For each triazole product, a calibration curve was performed to convert fluorescence intensity signal into product conversion (Figures S4–S6). Of note, the presence of copper(I) did not significantly modify the fluorescence emission of either triazole products **9**–**11**.

**Figure 3:**
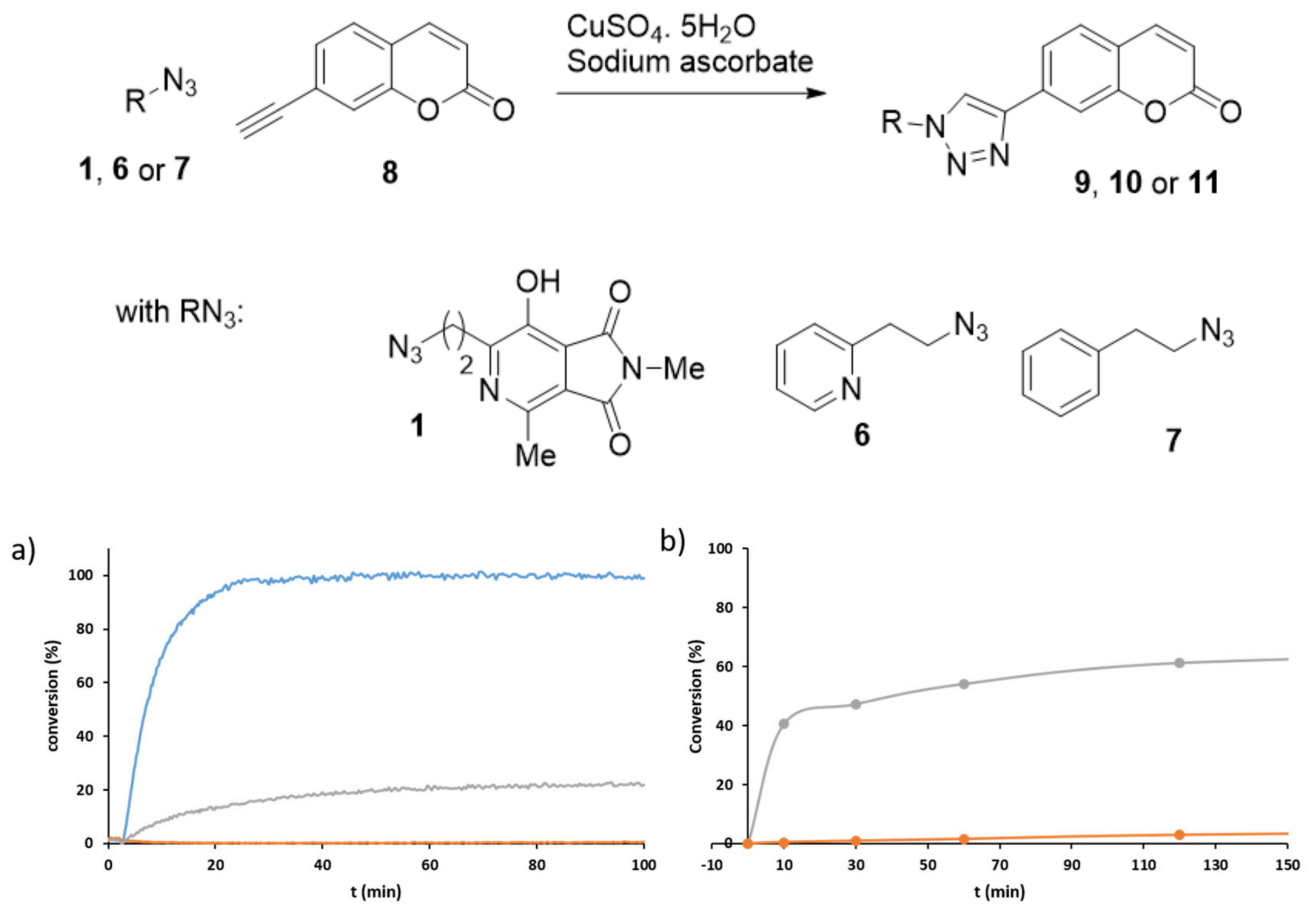
Comparison of kinetics of CuAAC reactions of: (**a**) 7-ethynyl coumarin (17.5 μM), with azide (**1** in grey, **6** in blue, and **7** in orange) in the presence of CuSO_4_·5H_2_0 (17.5 μM) and sodium ascorbate (437 μM) in DMF:PBS pH 7.4:H_2_O (1:1:2); (**b**) 7-ethynyl coumarin (175 μM), with azide (**1** in grey, and **7** in orange) in the presence of CuSO_4_.5H_2_O (175 μM) and sodium ascorbate (4.375 μM) in DMF:PBS pH 7.4:H_2_O (1:1:2).

First, azide **1**, **6,** and **11** were reacted with coumarin **8** and CuSO_4_.5 H2O (17.5 μM each), followed by the addition of sodium ascorbate (0.437 mM, 25 equiv.) in aqueous medium (Figure 3a).

First, the 2-(2-azidoethyl)-pyridine **6** led to the rapid formation of the triazole product (Figure 3a, curve in blue), while on the other hand, the non-chelating azide **7** gave no detectable product, even after 100 min of reaction (curve in orange). Meanwhile, the azaphthalimide enables the formation of 20% triazole. Although this conversion is significantly lower than that of **6**, this result showed that the azapthalimide was able to accelerate the reaction, presumably by acting as a bidentate ligand for CuAAC reactions. This chelating ability has been further validated with reactions carried out at 175 μM, and monitored by HPLC (Figure 2b, grey curve). In fact, a plateau at ca. 60% conversion yield was quickly reached, while almost no product was obtained with the non-chelating compound (orange curve). The reaction performed with the azaphthalimide was approximatively 100 times faster than the one achieved with the phenyl scaffold. Importantly, the presence of the copper complex did not impact the absorption and emission wavelengths of either fluorophores (Figure S1).

### 3.3. Applications

**Scheme 2:**
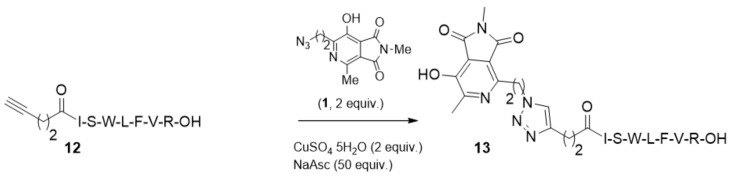
Labeling of alkyne-oligopeptide **12** with azide **1**.

The potency of a small fluorophore-assisted click chemistry for minimal conjugate perturbation was illustrated through the fluorescent labeling of a heptapeptide-based short biomolecule (Scheme 2). The starting peptide was prepared according to standard solid-phase peptide synthesis protocols with the incorporation of the pentynoic acid at the N-terminus (see Materials and Methods).

**Figure 4:**
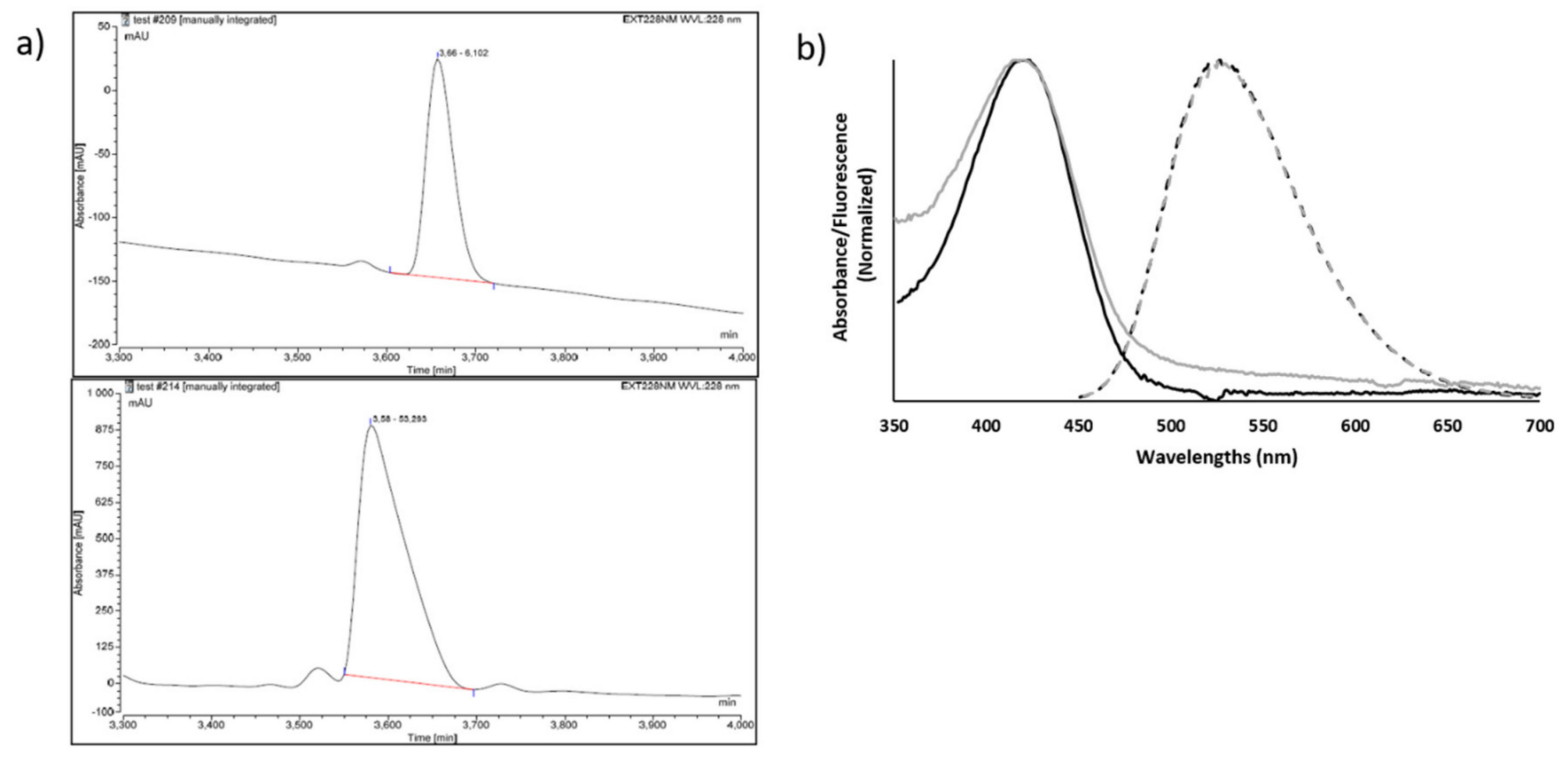
Green-fluorescence labeling of peptide **12**. (**a**) RP-HPLC chromatogram of peptide-alkyne **12** (top) and labeled peptide **13** after 30 min reaction (bottom). (**b**) Normalized absorption (solid line) and emission (dashed line) spectra of azaphthalimide-containing peptide **13** (in black) in the presence of in situ generated copper(I) (grey line) in PBS pH 7.4 at 20 °C.

The reaction was monitored by HPLC and showed a clean and complete reaction within 30 min (Figure 4a). The isolated conjugate **13** obtained in a 38% isolated yield after RP-HPLC and lyophilization was subject to photophysical measurements. Absorption and emission spectra recorded either in the presence or absence of copper(I) (generated in situ from CuSO_4_·5H_2_O and sodium ascorbate), showed no change in positions of peak maxima, which were found at λ_abs_ ~ 416 nm and λ_em_ ~ 525 nm, respectively (Figure 4b). Only a widening of the absorption peak was observed. Besides, quantum yield for the green fluorescence-labeled peptide was found to be around 8% in the presence and absence of in situ generated copper(I) determined in PBS pH 7.4 at 25 °C. This value is in line with those reported for similar azaphthalimide derivatives [29].

**Figure 5:**
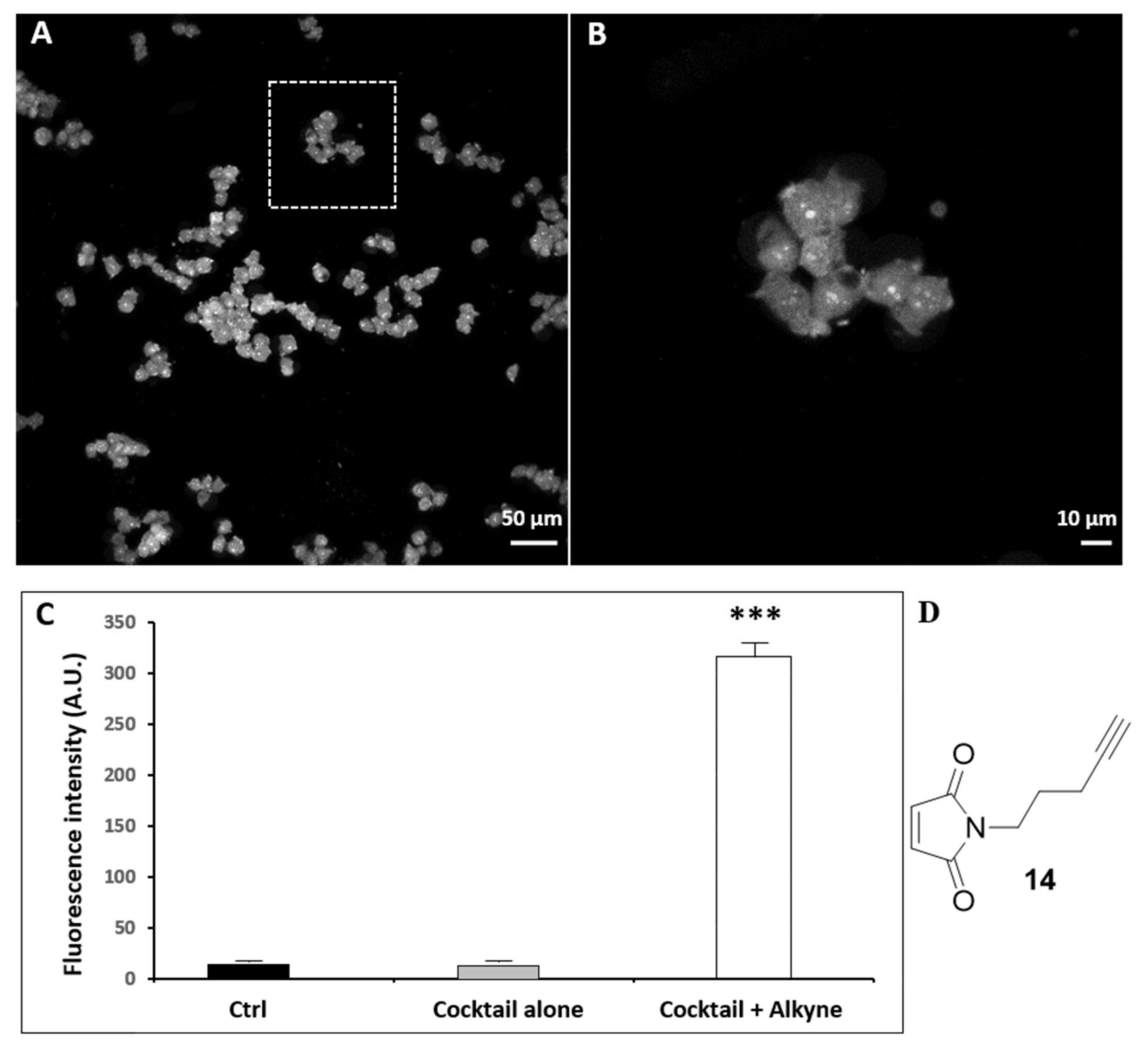
(**A**,**B**) PC12 cells in «Cocktail+Alkyne» conditions, acquired under mozaic confocal image for cell population observation (**A**) and single images for cellular observation (**B**). (**C**) Fluorescence intensity graph showing high significance (*** *p* < 0.001) of the fluorescence intensity level after «Cocktail+Alkyne» incubation *versus* «cocktail alone» and PBS incubation (ctrl). (**D**) Chemical structure of alkynyl maleimide **14**.

After establishing that small conjugates could be readily labeled in vitro using a heptapeptide as the model, then the labeling of alkyne-modified live cells with azaphthalimide azide **1** was investigated. As a proof-of-concept study, the alkyne reporter group was first chemically incorporated into live cells by using a maleimide derivative **14** (50 μM in PBS for 30 min at 37 °C), a functional group known to react rapidly and covalently with biological thiols through a Michael addition process [35]. Cells were washed twice with PBS to remove unreacted alkyne-maleimide **14** in the supernatant; then, a 50 μM solution of azaphthalimide-based chelating azide **1** in PBS pretreated with a 50 μM solution of in situ generated copper(I) (cocktail solution) was incubated for 15 min with cells at 37 °C, and then rinsed with PBS. Subsequent confocal analysis microscopy revealed strong fluorescence labeling of cells at 500–550 nm (Figure 5)—highly statistically significant compared to control conditions (fluorescence poorly detectable in cells with only PBS incubation (ctrl) and “cocktail alone,” when cells that were not incubated with the alkyne reporter group, thereby demonstrating successful CuAAC-mediated ligation of azaphthalimide azide and alkyne incorporated into cells, data not shown). Images revealed some discernible vesicles that could be identified as lipid droplets, in which the lipophilic alkyne reporter presumably accumulates (Figure 5A,B and Figure S7) [36].

## 4. Conclusions

Herein, we disclosed an unprecedented “all-in-one” fluorophore-based chelating azide with an exceptionally low molecular weight (260 Da), which proved useful for the fluorescent labeling of small biomolecules and biological systems. In this approach, the azaphthalimide played the role of both the copper ligand and fluorophore in order to accelerate the CuAAC reaction with concomitant installation of a fluorescent tag. Importantly, the fluorescence properties of the native fluorophore were conserved upon its complexation to cooper(I). This process advantageously does not require an external ligand, a fluorophore, or water solubilizing group, which complicates the synthesis and increases the risk of impacting the mobility of azides and properties of conjugated molecules. This strategy could be extended to other families of nitrogen-containing fluorophores in order to further improve the ligation rates or to red-shift the fluorescence emission [37,38].

## Supplementary Materials

The following are available online at https://www.mdpi.com/2218-273X/10/4/619/s1. ^1^H and ^13^C NMR spectra for all new compounds; absorption, emission, and excitation spectra for compounds **9**–**11**; additional information regarding live cell labeling.

## References

[1] Rostovtsev V.V., Green L.G., Fokin V.V., Sharpless K.B. (2002). A Stepwise Huisgen Cycloaddition Process: Copper(I)-Catalyzed Regioselective “Ligation” of Azides and Terminal Alkynes. Angew. Chem. Int. Ed..

[2] Tornøe C.W., Christensen C., Meldal M. (2002). Peptidotriazoles on Solid Phase:  [1,2,3]-Triazoles by Regiospecific Copper(I)-Catalyzed 1,3-Dipolar Cycloadditions of Terminal Alkynes to Azides. J. Org. Chem..

[3] Bruyat P., Gautier A., Jean L., Renard P.-Y. (2018). Use of an Air-Stable Cu(I)-NHC Catalyst for the Synthesis of Peptidotriazoles. J. Org. Chem..

[4] Farzan V.M., Ulashchik E.A., Martynenko-Makaev Y.V., Kvach M.V., Aparin I.O., Brylev V.A., Prikazchikova T.A., Maklakova S.Y., Majouga A.G., Ustinov A.V. (2017). Automated Solid-Phase Click Synthesis of Oligonucleotide Conjugates: From Small Molecules to Diverse N-Acetylgalactosamine Clusters. Bioconjugate Chem..

[5] Simon C., Lion C., Spriet C., Baldacci-Cresp F., Hawkins S., Biot C. (2018). One, Two, Three: A Bioorthogonal Triple Labelling Strategy for Studying the Dynamics of Plant Cell Wall Formation In Vivo. Angew. Chem. Int. Ed..

[6] Jiang H., English B.P., Hazan R.B., Wu P., Ovryn B. (2015). Tracking Surface Glycans on Live Cancer Cells with Single-Molecule Sensitivity. Angew. Chem. Int. Ed..

[7] Mas Pons J., Dumont A., Sautejeau G., Fugier E., Baron A., Dukan S., Vauzeilles B. (2014). Identification of Living Legionella pneumophila Using Species-Specific Metabolic Lipopolysaccharide Labeling. Angew. Chem. Int. Ed..

[8] Yuan Y., Xu S., Cheng X., Cai X., Liu B. (2016). Bioorthogonal Turn-On Probe Based on Aggregation-Induced Emission Characteristics for Cancer Cell Imaging and Ablation. Angew. Chem. Int. Ed..

[9] Haldón E., Nicasio M.C., Pérez P.J. (2015). Copper-catalysed azide–alkyne cycloadditions (CuAAC): An update. Org. Biomol. Chem..

[10] Li L., Zhang Z. (2016). Development and Applications of the Copper-Catalyzed Azide-Alkyne Cycloaddition (CuAAC) as a Bioorthogonal Reaction. Molecules.

[11] Kenry, Liu B. (2019). Bio-orthogonal Click Chemistry for in vivo Bioimaging. Trends Chem..

[12] Yang M., Li J., Chen P.R. (2014). Transition metal-mediated bioorthogonal protein chemistry in living cells. Chem. Soc. Rev..

[13] Agard N.J., Prescher J.A., Bertozzi C.R. (2004). A Strain-Promoted [3 + 2] Azide−Alkyne Cycloaddition for Covalent Modification of Biomolecules in Living Systems. J. Am. Chem. Soc..

[14] Blackman M.L., Royzen M., Fox J.M. (2008). Tetrazine Ligation: Fast Bioconjugation Based on Inverse-Electron-Demand Diels−Alder Reactivity. J. Am. Chem. Soc..

[15] Devaraj N.K., Weissleder R., Hilderbrand S.A. (2008). Tetrazine-Based Cycloadditions: Application to Pretargeted Live Cell Imaging. Bioconjugate Chem..

[16] Lee K.J., Kang D., Park H.S. (2019). Site-Specific Labeling of Proteins Using Unnatural Amino Acids. Mol. Cells.

[17] Hong V., Presolski S.I., Ma C., Finn M.G. (2009). Analysis and Optimization of Copper-Catalyzed Azide–Alkyne Cycloaddition for Bioconjugation. Angew. Chem..

[18] Besanceney-Webler C., Jiang H., Zheng T., Feng L., Soriano del Amo D., Wang W., Klivansky L.M., Marlow F.L., Liu Y., Wu P. (2011). Increasing the Efficacy of Bioorthogonal Click Reactions for Bioconjugation: A Comparative Study. Angew. Chem. Int. Ed..

[19] Rodionov V.O., Presolski S.I., Gardinier S., Lim Y.-H., Finn M.G. (2007). Benzimidazole and Related Ligands for Cu-Catalyzed Azide−Alkyne Cycloaddition. J. Am. Chem. Soc..

[20] Jones L.H., Beal D., Selby M.D., Everson O., Burslem G.M., Dodd P., Millbank J., Tran T.-D., Wakenhut F., Graham E.J.S. (2011). In-cell click labelling of small molecules to determine subcellular localisation. J. Chem. Biol..

[21] Li S., Wang L., Yu F., Zhu Z., Shobaki D., Chen H., Wang M., Wang J., Qin G., Erasquin U.J. (2017). Copper-catalyzed click reaction on/in live cells. Chem. Sci..

[22] Kuang G.-C., Guha P.M., Brotherton W.S., Simmons J.T., Stankee L.A., Nguyen B.T., Clark R.J., Zhu L. (2011). Experimental Investigation on the Mechanism of Chelation-Assisted, Copper(II) Acetate-Accelerated Azide–Alkyne Cycloaddition. J. Am. Chem. Soc..

[23] Brotherton W.S., Michaels H.A., Simmons J.T., Clark R.J., Dalal N.S., Zhu L. (2009). Apparent Copper(II)-Accelerated Azide−Alkyne Cycloaddition. Org. Lett..

[24] Uttamapinant C., Tangpeerachaikul A., Grecian S., Clarke S., Singh U., Slade P., Gee K.R., Ting A.Y. (2012). Fast, Cell-Compatible Click Chemistry with Copper-Chelating Azides for Biomolecular Labeling. Angew. Chem. Int. Ed..

[25] Bevilacqua V., King M., Chaumontet M., Nothisen M., Gabillet S., Buisson D., Puente C., Wagner A., Taran F. (2014). Copper-Chelating Azides for Efficient Click Conjugation Reactions in Complex Media. Angew. Chem. Int. Ed..

[26] Sallustrau A., Bregant S., Chollet C., Audisio D., Taran F. (2017). Scalable and practical synthesis of clickable Cu-chelating azides. Chem. Commun..

[27] Birch D., Christensen M.V., Staerk D., Franzyk H., Nielsen H.M. (2017). Fluorophore labeling of a cell-penetrating peptide induces differential effects on its cellular distribution and affects cell viability. BBA Biomembranes.

[28] Jouanno L.A., Chevalier A., Sekkat N., Perzo N., Castel H., Romieu A., Lange N., Sabot C., Renard P.Y. (2014). Kondrat’eva ligation: Diels-Alder-based irreversible reaction for bioconjugation. J. Org. Chem..

[29] Renault K., Jouanno L.-A., Lizzul-Jurse A., Renard P.-Y., Sabot C. (2016). Fluorogenic Behaviour of the Hetero-Diels–Alder Ligation of 5-Alkoxyoxazoles with Maleimides and their Applications. Chem. Eur. J..

[30] Stewart W.W. (1981). Synthesis of 3,6-disulfonated 4-aminonaphthalimides. J. Am. Chem. Soc..

[31] Li M., Dong K., Zheng Y., Song W. (2019). Copper-catalyzed cascade click/nucleophilic substitution reaction to access fully substituted triazolyl-organosulfurs. Org. Biomol. Chem..

[32] Nunes J.P.M., Morais M., Vassileva V., Robinson E., Rajkumar V.S., Smith M.E.B., Pedley R.B., Caddick S., Baker J.R., Chudasama V. (2015). Functional native disulfide bridging enables delivery of a potent, stable and targeted antibody–drug conjugate (ADC). Chem. Commun..

[33] Jouanno L.A., Sabot C., Renard P.Y. (2012). Expeditious microwave-assisted synthesis of 5-alkoxyoxazoles from alpha-triflyloxy esters and nitriles. J. Org. Chem..

[34] Gude M., Ryf J., White P.D. (2002). An accurate method for the quantitation of Fmoc-derivatized solid phase supports. J. Pept. Sci..

[35] Renault K., Fredy J.W., Renard P.-Y., Sabot C. (2018). Covalent Modification of Biomolecules through Maleimide-Based Labeling Strategies. Bioconjugate Chem..

[36] Cohen S. (2018). Lipid Droplets as Organelles. Int. Rev. Cell Mol. Biol..

[37] Atkins R.L., Bliss D.E. (1978). Substituted coumarins and azacoumarins. Synthesis and fluorescent properties. J. Org. Chem..

[38] Takano H., Narumi T., Nomura W., Tamamura H. (2017). Microwave-Assisted Synthesis of Azacoumarin Fluorophores and the Fluorescence Characterization. J. Org. Chem..

