# Supplementary material for: Fluorophore-Assisted Click Chemistry through Copper(I) Complexation
Source: Biomolecules. 2020 Apr 16;10(4):619. doi: 10.3390/biom10040619 (PMC7225998; doi:10.3390/biom10040619)
Supplement: Supplementary file 1 [file biomolecules-10-00619-s001.pdf]

Supporting Information

Fluorophore-assisted click chemistry through copper(I) complexation

Victor Flon, Magalie Bénard, Damien Schapman, Ludovic Galas, Pr. Pierre-Yves Renard and Dr. Cyrille Sabot*

<cyrille.sabot@univ-rouen.fr>

Normandie Univ, Laboratoire COBRA UMR 6014, Univ Rouen-Normandie and CNRS, 1 rue Lucien Tesnière 76821 Mont-Saint-Aignan, Cedex, France.

Table of Contents

I. Copies of ^{1}H and ^{13}C NMR 2
II. Absorption, emission data for compounds **9-11**: 9
III. Kinetic studies: 11
III.1. For azides, alkynes and copper sulfate at the concentration of 175 µM 11
III.2. For azides, alkynes and copper sulfate at the concentration of 17.5 µM 11
III.3. Calibration Curves 12
IV. Live cells labeling: 13
IV.1. Preparation of stock solutions 13
IV.2. Complementary confocal microscopy analysis: 14

I. Copies of ^{1}H and ^{13}C NMR

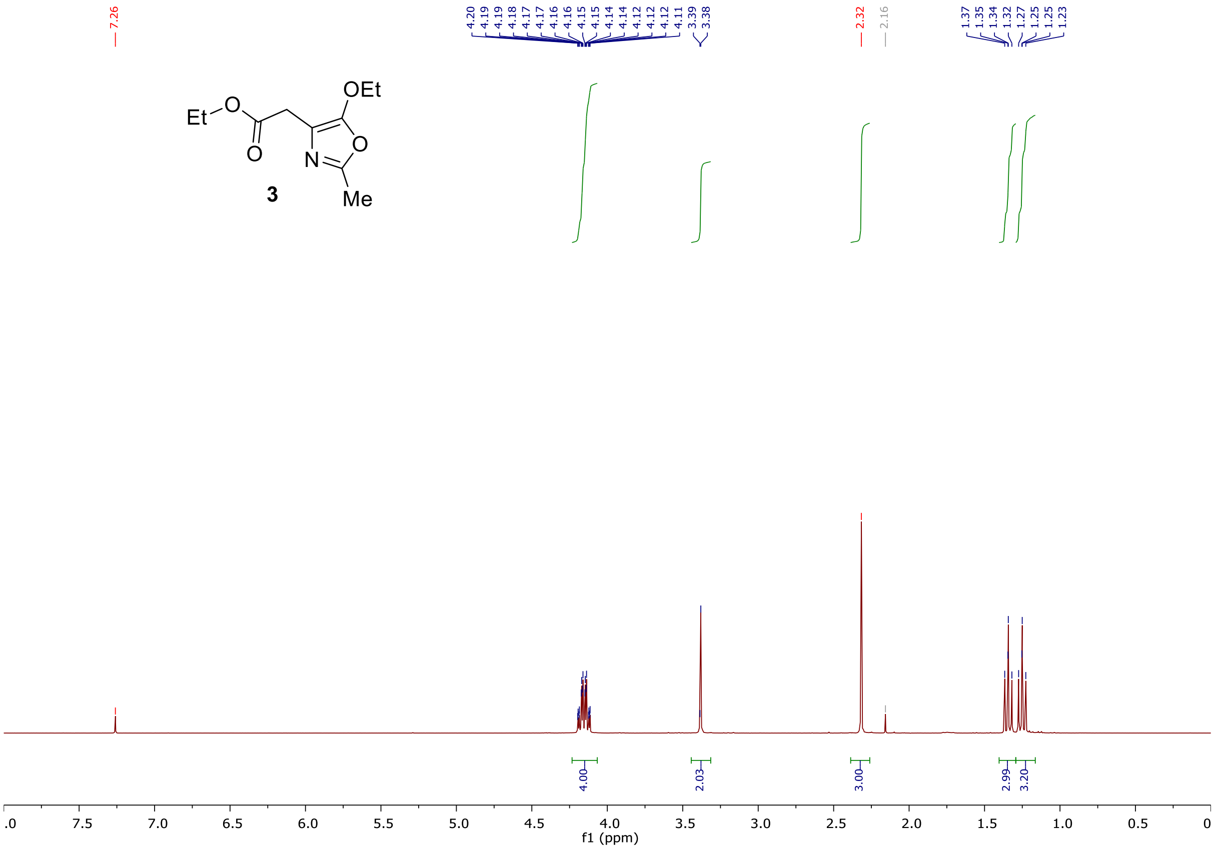

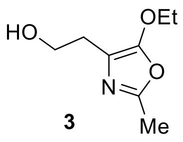
HO
OEt
O
N
3
Me

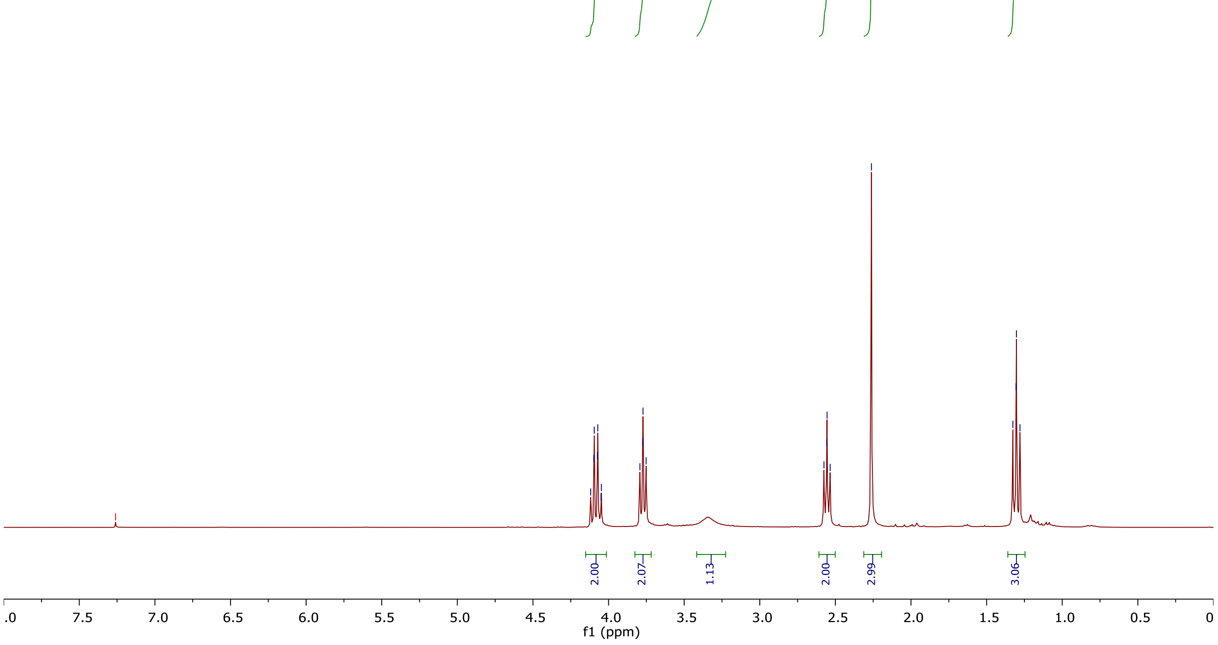
7.26 CDCl3
4.12
4.10
4.09
4.07
4.07
4.05
4.05
3.79
3.77
3.77
3.75
2.58
2.56
2.55
2.53
2.26
1.33
1.31
1.30
1.28
2.00
2.07
1.13
2.00
2.99
3.06
f1 (ppm)

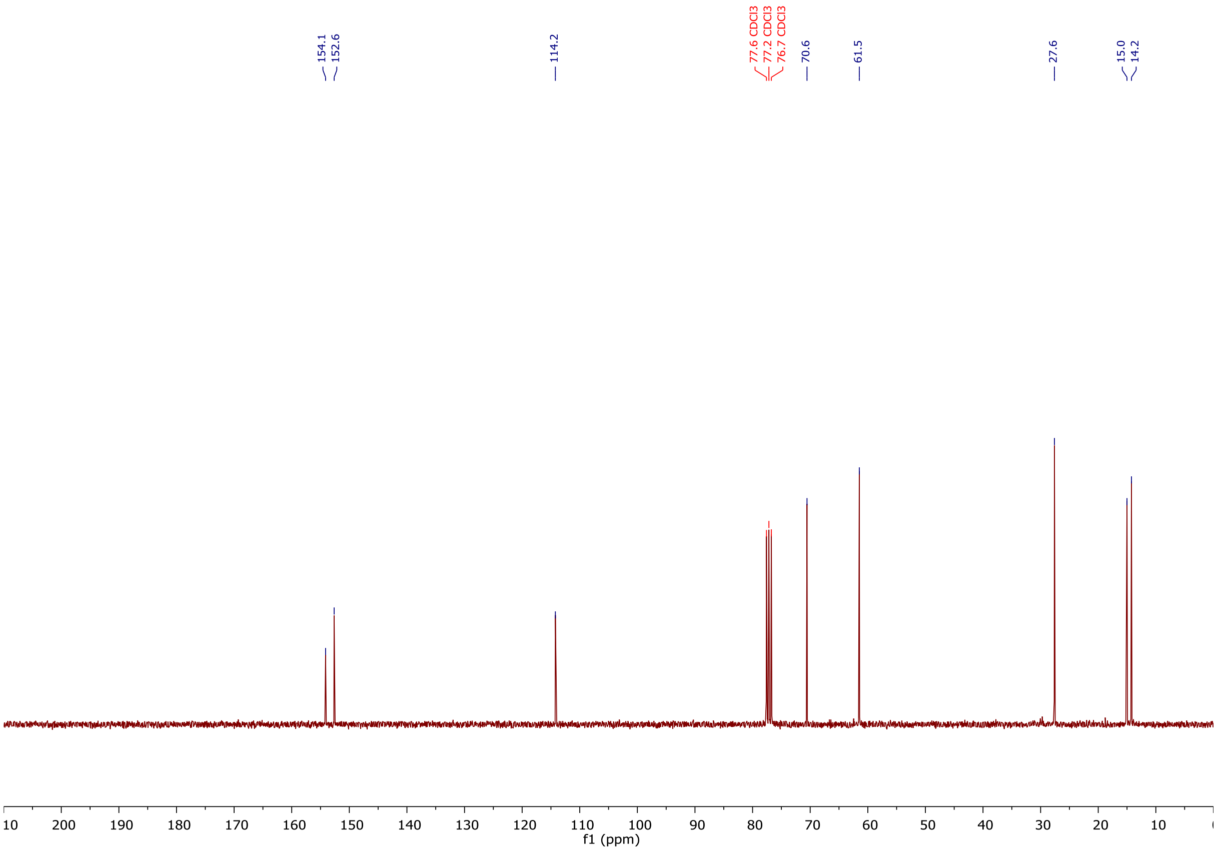
154.1
152.6
114.2
77.6 CDCl3
77.2 CDCl3
76.7 CDCl3
70.6
61.5
27.6
15.0
14.2
f1 (ppm)

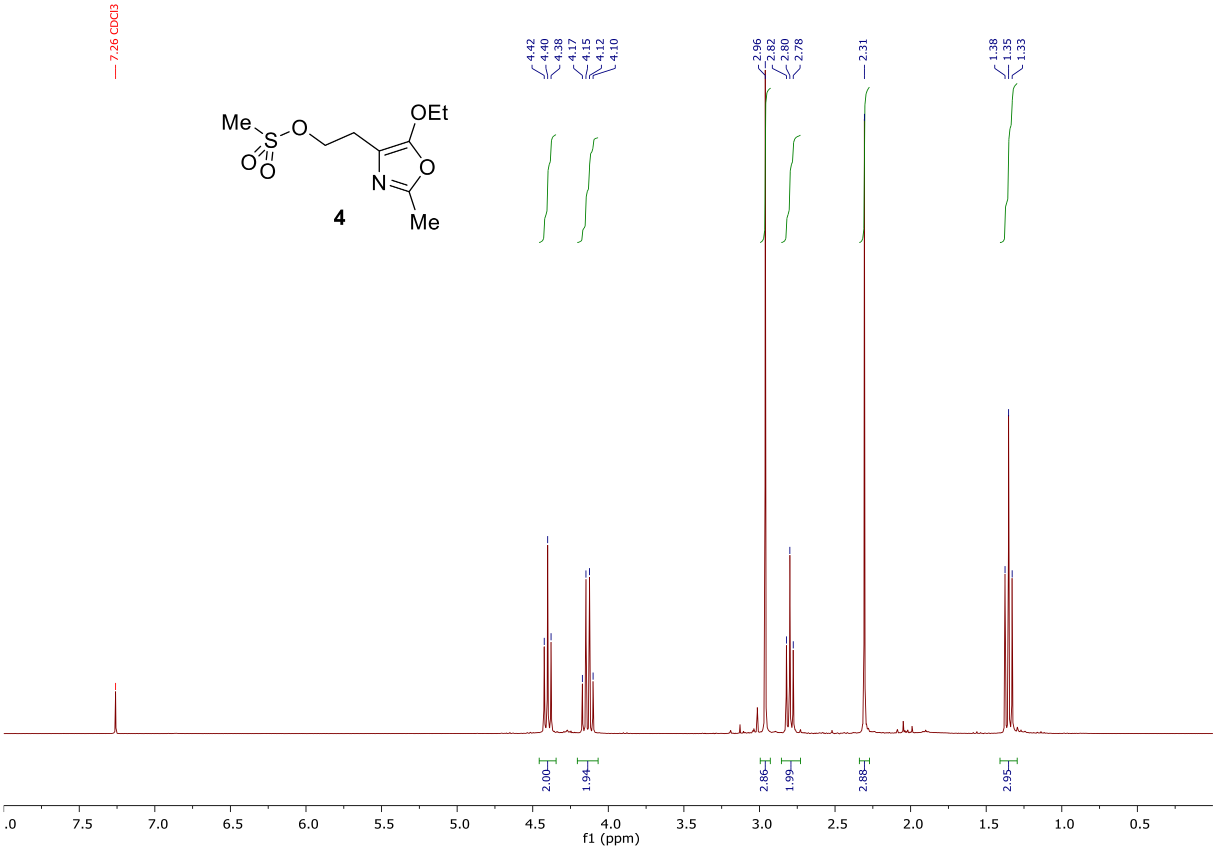
7.26 CDCl3
4.42
4.40
4.38
4.17
4.15
4.12
4.10
2.96
2.82
2.80
2.78
2.31
1.38
1.35
1.33
OEt
Me
S
O
O
O
N
O
Me
4
2.00
1.94
2.86
1.99
2.88
2.95
f1 (ppm)

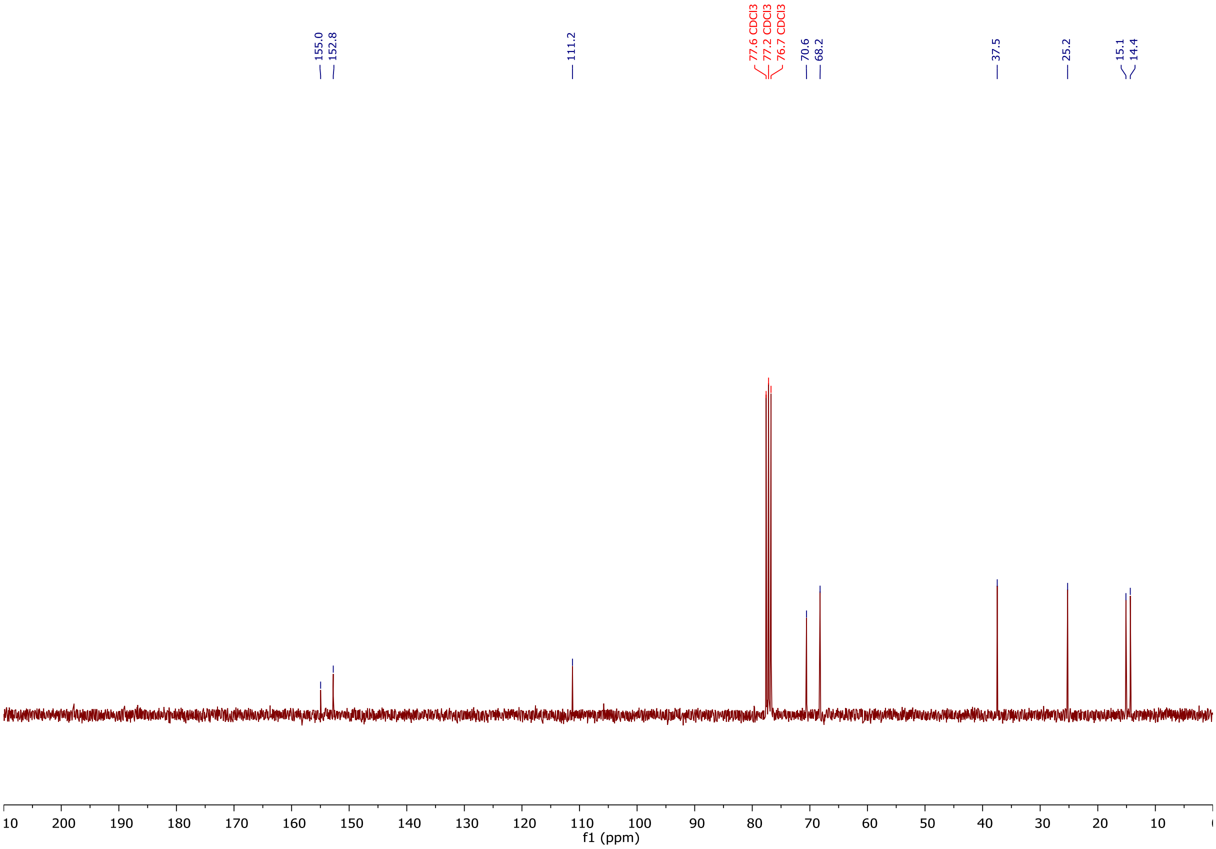
155.0
152.8
111.2
77.6 CDCl3
77.2 CDCl3
76.7 CDCl3
70.6
68.2
37.5
25.2
15.1
14.4
f1 (ppm)

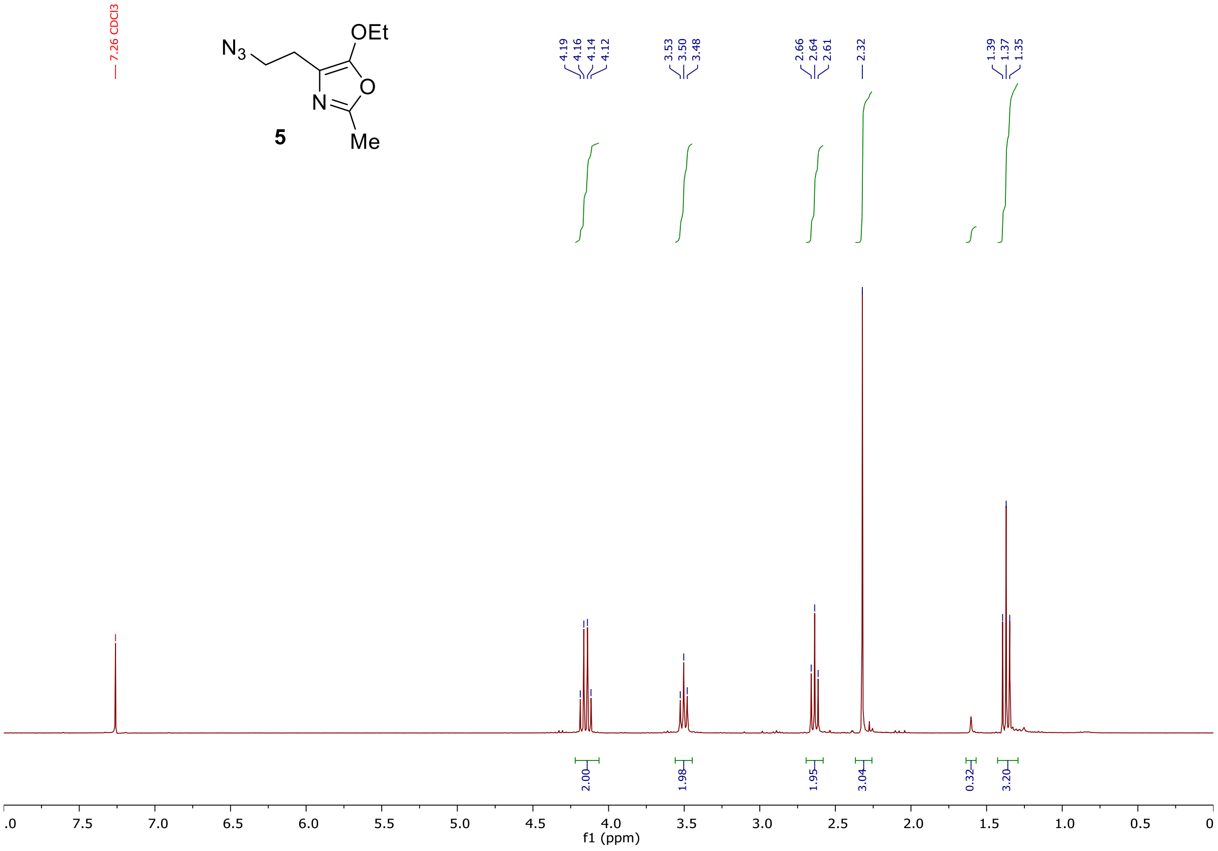
7.26 CDCl3
N3
OEt
O
N
Me
5
4.19
4.16
4.14
4.12
3.53
3.50
3.48
2.66
2.64
2.61
2.32
1.39
1.37
1.35
2.00
1.98
1.95
3.04
0.32
3.20
f1 (ppm)

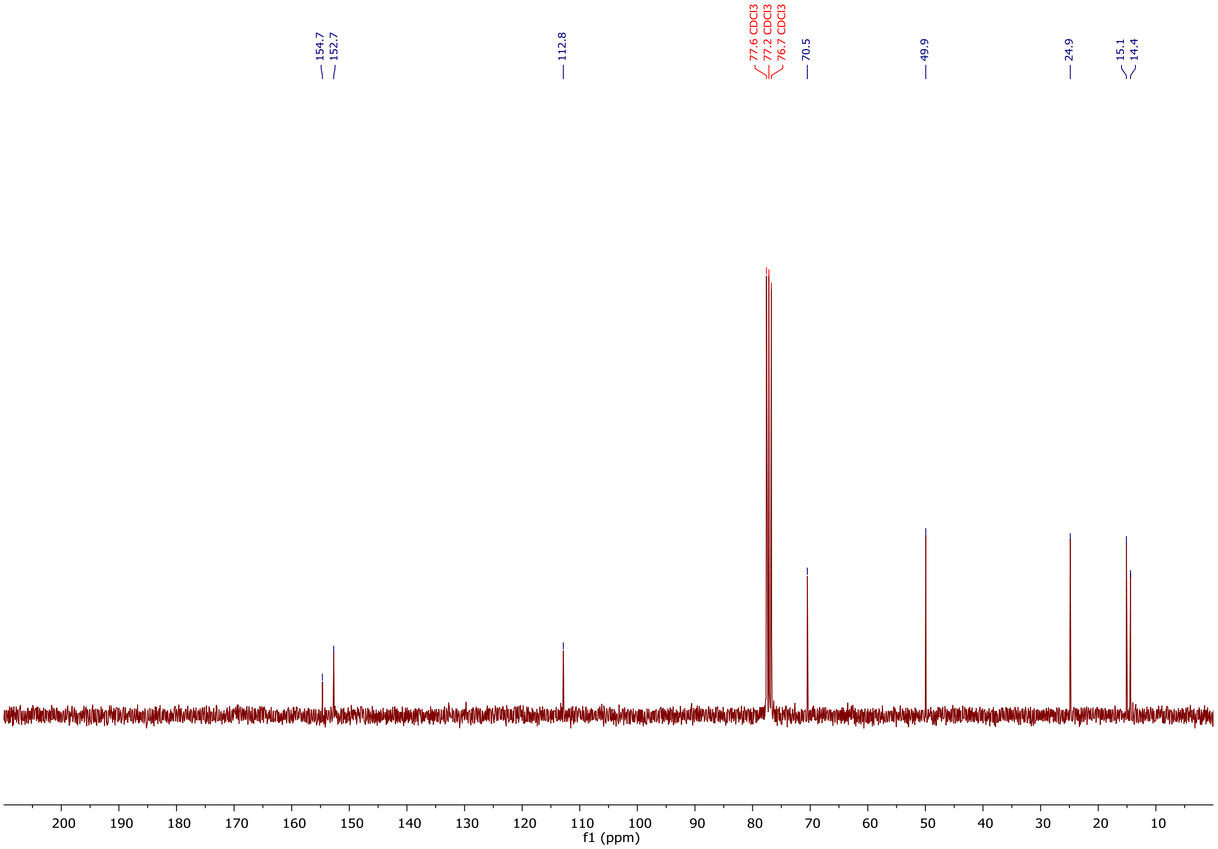
154.7
152.7
112.8
77.6 CDCl3
77.2 CDCl3
76.7 CDCl3
70.5
49.9
24.9
15.1
14.4
f1 (ppm)

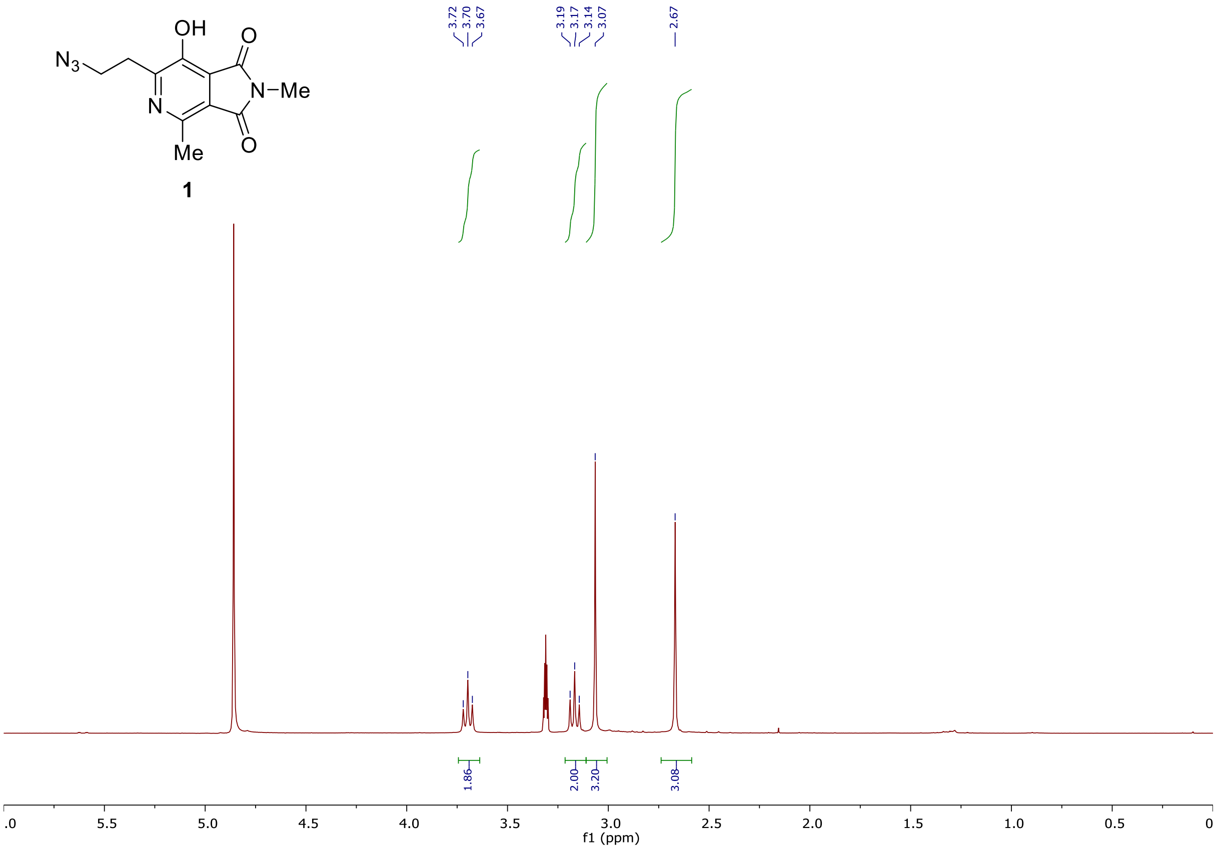
OH
O
N₃
N−Me
N
Me
O
1
3.72
3.70
3.67
3.19
3.17
3.14
3.07
2.67
1.86
2.00
3.20
3.08
f1 (ppm)

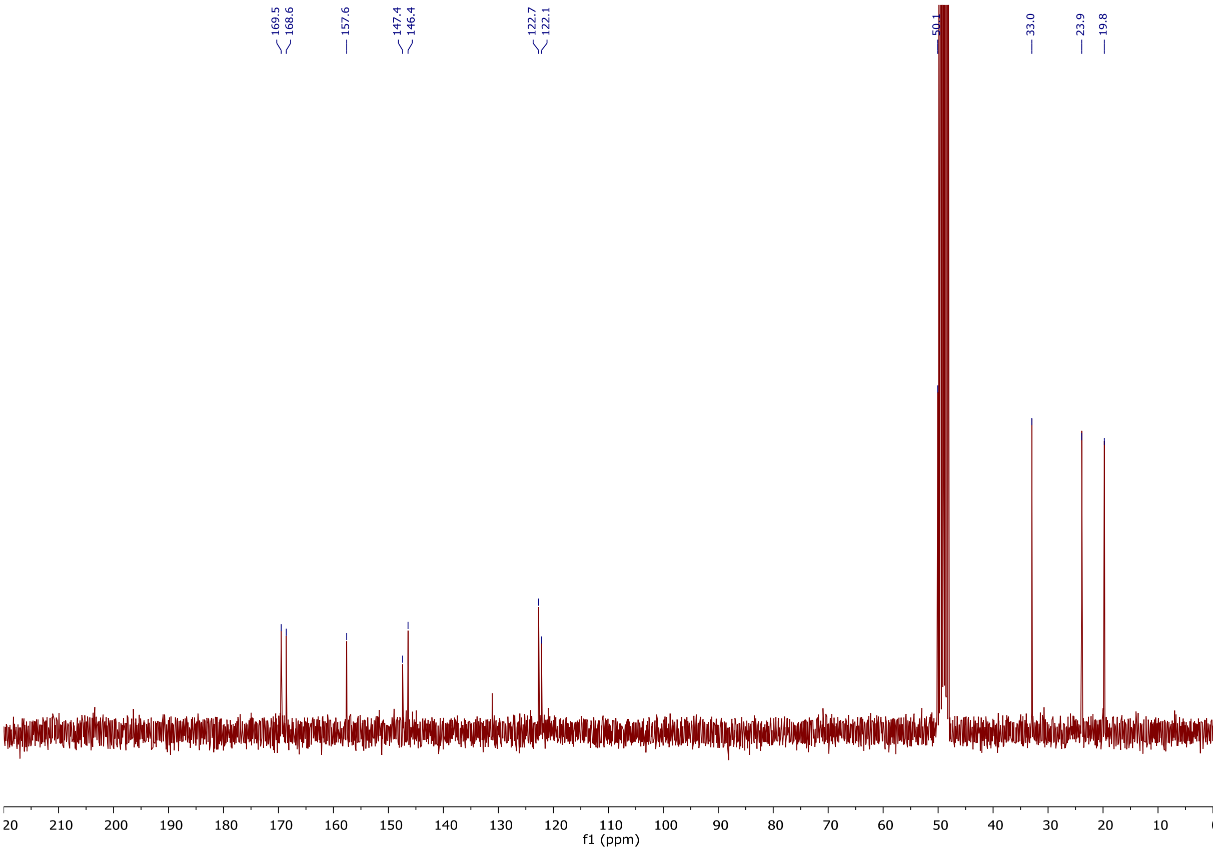
169.5
168.6
157.6
147.4
146.4
122.7
122.1
50.1
33.0
23.9
19.8
f1 (ppm)

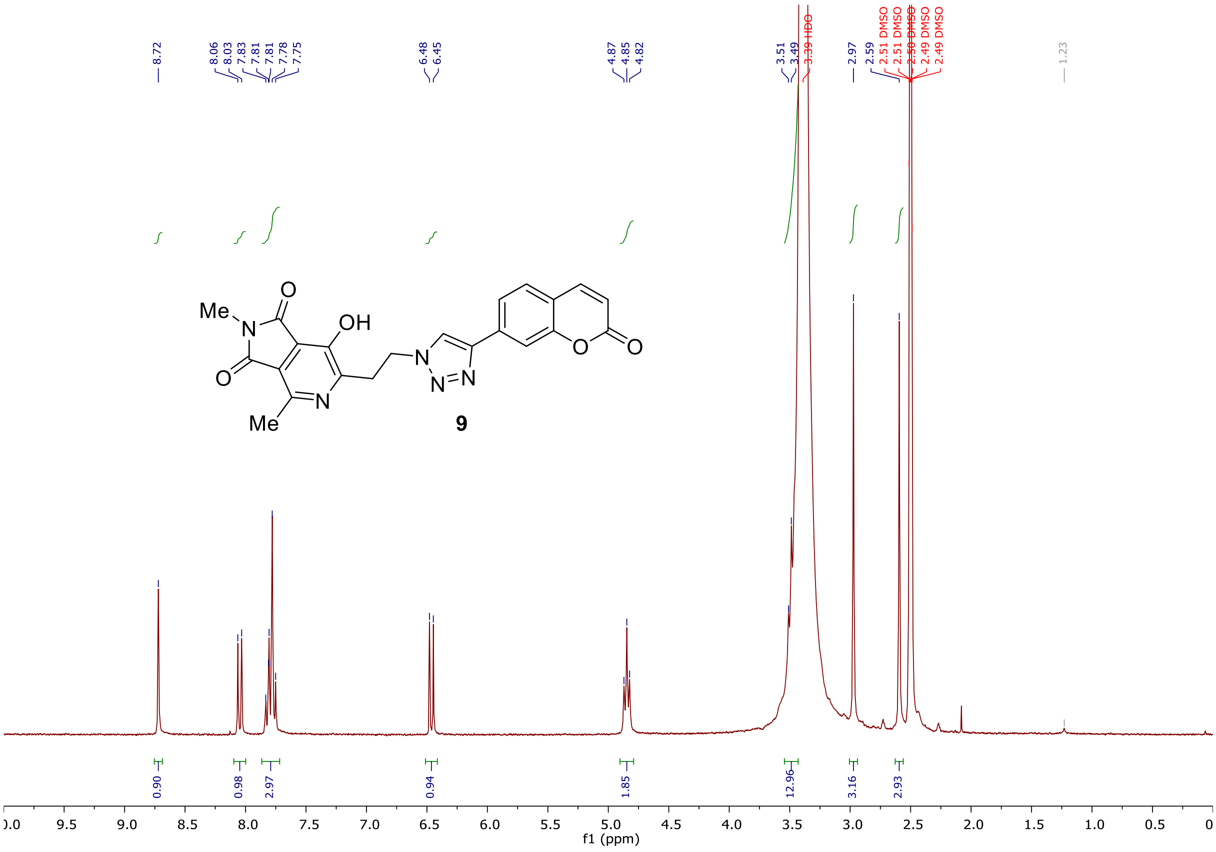
8.72
8.06 8.03 7.83 7.81 7.81 7.78 7.75
6.48 6.45
4.87 4.85 4.82
3.51 3.49 3.39 HDO
2.97 2.59 2.51 DMSO 2.51 DMSO 2.50 DMSO 2.49 DMSO 2.49 DMSO
1.23
Me N O O OH Me N N N N O O
9
0.90 0.98 2.97 0.94 1.85 12.96 3.16 2.93
f1 (ppm)

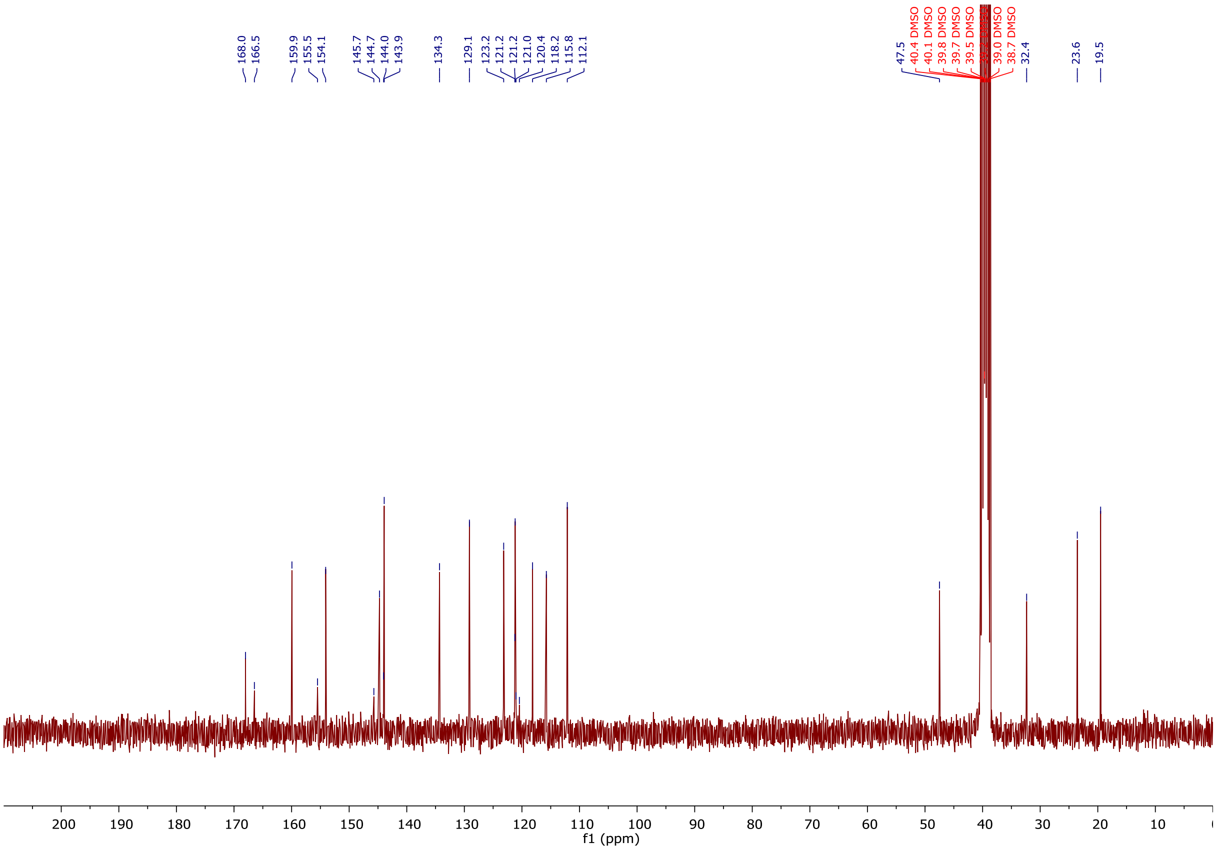
168.0 166.5 159.9 155.5 154.1 145.7 144.7 144.0 143.9 134.3 129.1 123.2 121.2 121.2 121.0 120.4 118.2 115.8 112.1
47.5 40.4 DMSO 40.1 DMSO 39.8 DMSO 39.7 DMSO 39.5 DMSO 39.0 DMSO 38.7 DMSO 32.4 23.6 19.5
f1 (ppm)

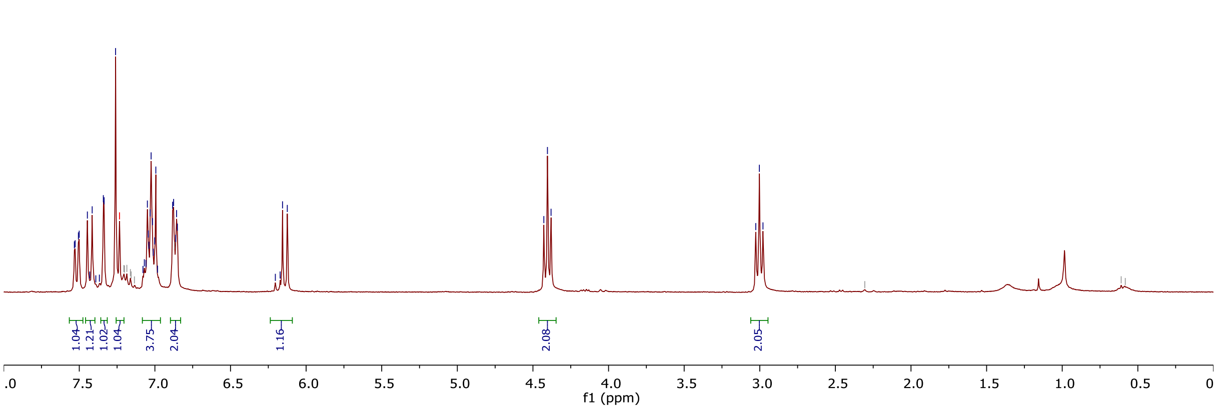
7.53 7.53 7.51 7.50 7.45 7.43 7.41 7.39 7.37 7.34 7.34 7.26 7.23 CDCl3 7.21 7.20 7.19 7.16 7.16 7.14 7.08 7.07 7.06 7.05 7.04 7.04 7.03 7.02 7.02 7.01 7.00 6.99 6.98 6.88 6.88 6.87 6.86 6.86 6.85 6.20 6.17 6.16 6.12 4.43 4.40 4.38
3.03 3.00 2.98
2.31
0.61 0.58
1.04 1.21 1.02 1.04 3.75 2.04 1.16 2.08 2.05
f1 (ppm)

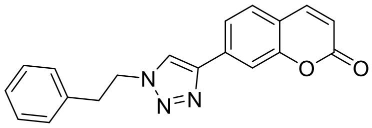
N
N
N
O
O

11

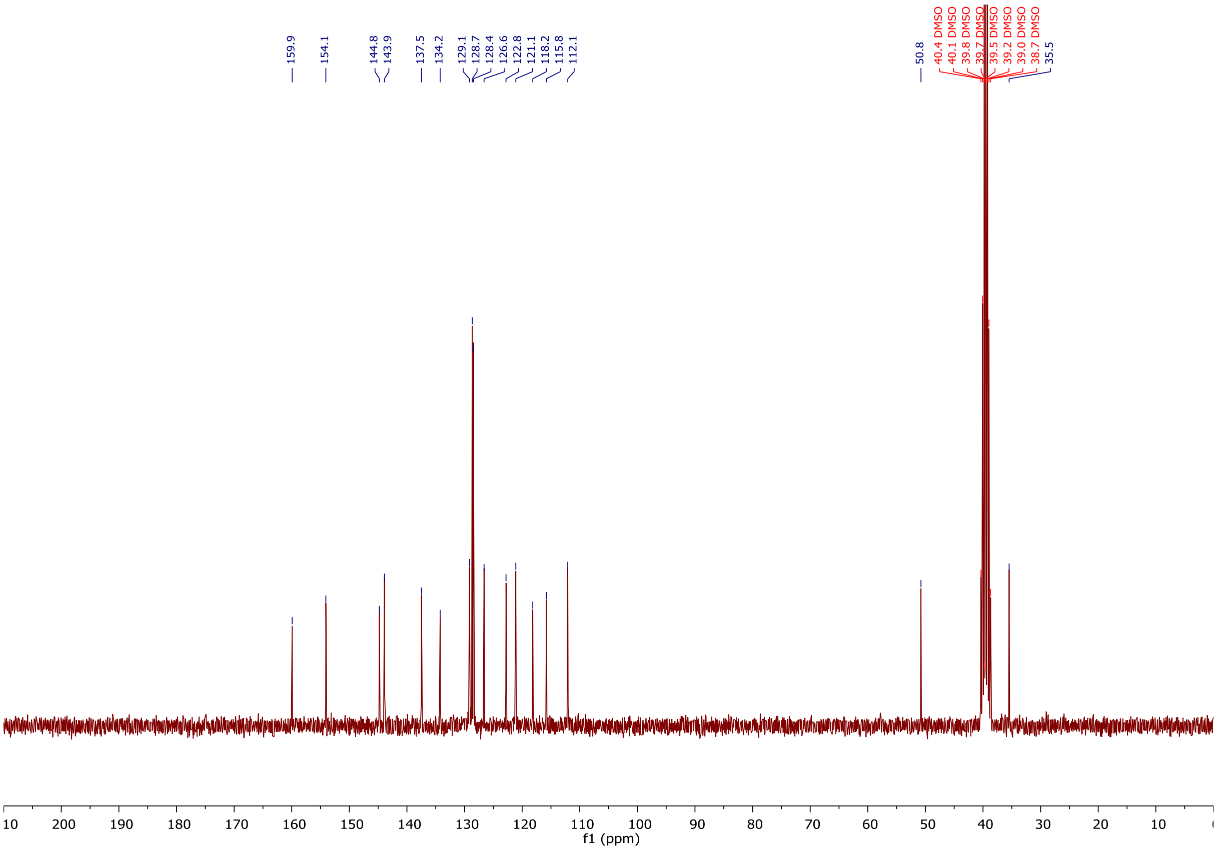
159.9
154.1
144.8 143.9
137.5 134.2
129.1 128.7 128.4 126.6 122.8 121.1 118.2 115.8 112.1
50.8
40.4 DMSO 40.1 DMSO 39.8 DMSO 39.7 DMSO 39.5 DMSO 39.2 DMSO 39.0 DMSO 38.7 DMSO
35.5
f1 (ppm)

II. Absorption, emission data for compounds **9-11**:

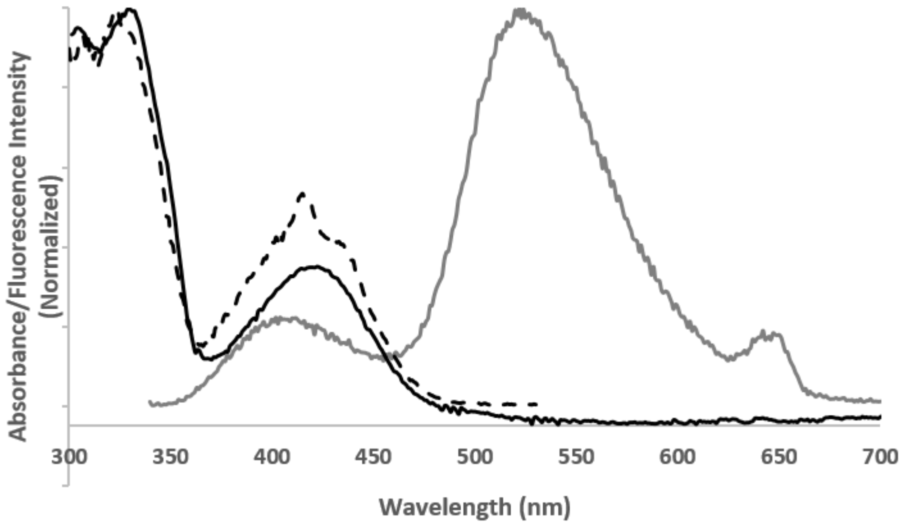

Figure S1. Normalized absorption (black line), emission (grey line, λ_{ex} = 320 nm), excitation (dashed line, λ_{em} = 550 nm) for triazole-azaphthalimide **9** in PBS pH 7.4 at 20 °C.

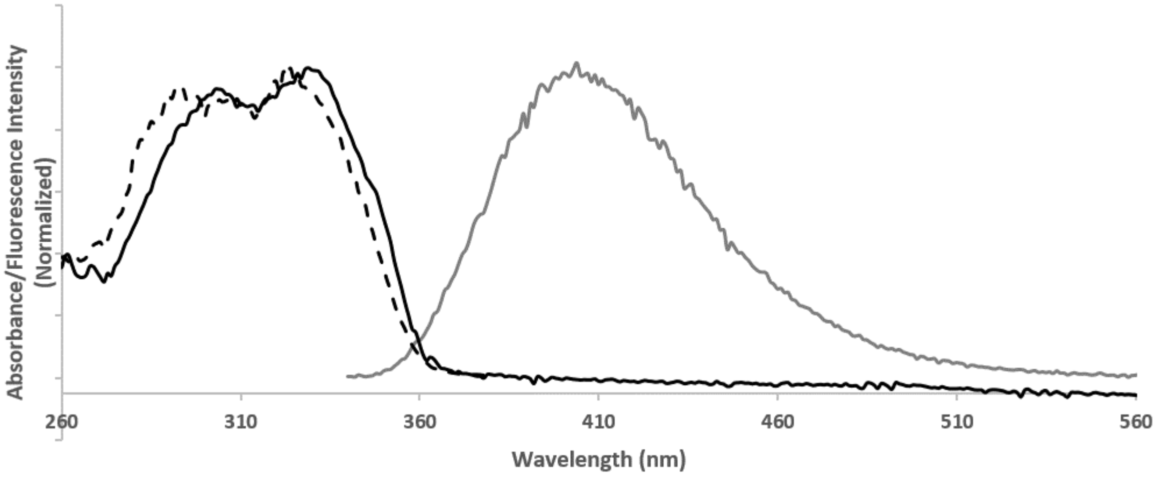

Figure S2. Normalized absorption (black line), emission (grey line, λ_{ex} = 320 nm), excitation (dashed line, λ_{em} = 400 nm) for triazole-pyridine **10** in PBS pH 7.4 at 20 °C.

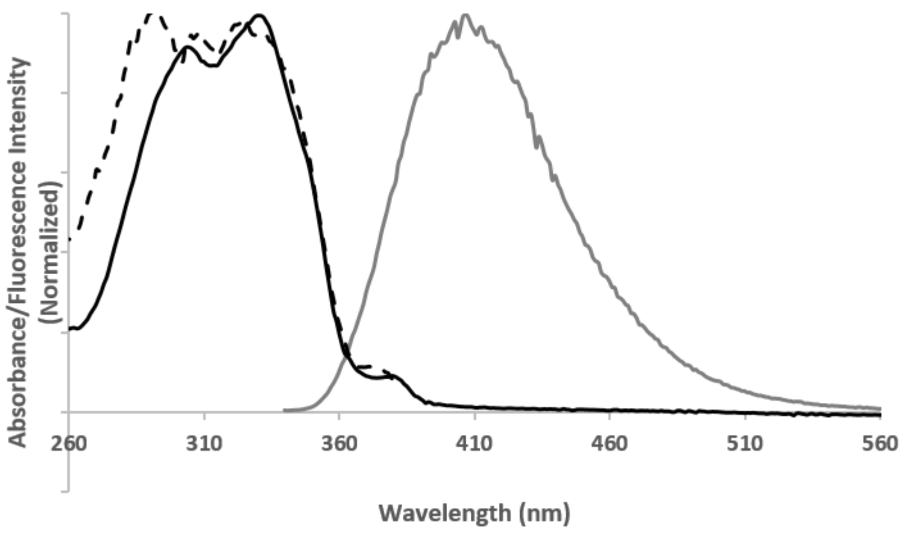

Figure S3. Normalized absorption (black line), emission (grey line, λ_{ex} = 320 nm), excitation (dashed line, λ_{em} = 400 nm) for triazole-phenyl **11** in PBS pH 7.4 at 20 °C.

III. Kinetic studies:

III.1. For azides, alkynes and copper sulfate at the concentration of 175 µM

Preparation of stock solutions:

- Copper sulfate pentahydrate: 700 µM in milliQ water (1 mL stock solution)
- Sodium ascorbate: 17.5 mM in milliQ water (1 mL stock solution)
- Solution of substrates (350 µM) was composed of:
    - 50 µL of a solution of azide at 6.25 mM in DMF
    - 50 µL of a solution of 7-ethynylcoumarin at 6.25 mM in DMF
    - DMF (340 µL)
    - PBS 0.1 M pH 7.4 (450 µL)

Procedure (final concentration of azide, alkyne and copper sulfate pentahydrate: 175 µM; and sodium ascorbate: 4.375 mM, 25 equiv.)

To a solution of copper sulfate pentahydrate (350 µL, 700 µM in milliQ water) in a 2-mL Eppendorf tube was added the solution of substrates (700 µL, 350 µM in DMF/PBS 0.1 M pH 7.4, ~1:1). After 30 min of incubation at 20 °C, the ascorbate solution (350 µL, 17.5 mM in milliQ water) was added to the mixture and the reaction was monitored by RP-HPLC at t = 10, 30, 60, 120, and 300 min. Preparation of samples for RP-HPLC analysis: to a 100 µL aliquot of the reaction mixture were successively added MeCN (100 µL) and milliQ water (300 µL). HPLC method A (λ_{Abs} = 300 nm for experiment with phenylethyl azide **7**, and 372 nm for experiment with azaphthalimide **1**).

III.2. For azides, alkynes and copper sulfate at the concentration of 17.5 µM

Preparation of stock solutions:

- Copper sulfate pentahydrate: 70 µM in milliQ water (1 mL stock solution)
- Sodium ascorbate: 1.75 mM in milliQ water (1 mL stock solution)
- Solution of substrates (35 µM) was composed of:
    - 50 µL of a solution of azide at 625 µM in DMF
    - 50 µL of a solution of 7-ethynylcoumarin at 625 µM in DMF
    - DMF (340 µL)
    - PBS 0.1 M pH 7.4 (450 µL)

Procedure (final concentration of azide, alkyne and copper sulfate pentahydrate: 17.5 µM; and sodium ascorbate: 437 µM, 25 equiv.)

To a solution of copper sulfate pentahydrate (350 µL, 70 µM in milliQ water) in a 1.4 mL fluorescence cuvette was added the solution of substrates (700 µL, 35 µM in DMF/PBS 0.1 M pH 7.4, ~1:1). After 30 min of incubation at 20 °C, the ascorbate solution (350 µL, 1.75 mM in milliQ water) was added to the mixture and the reaction was monitored by fluorescence spectroscopy (kinetics mode, excitation and

emission slit: 10 nm, T = 20 °C) over 100 min. The fluorescence intensity was continuously measured (λ_{Ex} = 320 nm and λ_{Em} = 550 nm with triazole **9**; λ_{Ex} = 320 nm and λ_{Em} = 400 nm with trialzoles **10** and **11**).

III.3. Calibration Curves

Calibration curve was established for each triazole product **9-11** to convert fluorescence intensity signal into product conversion.
Azaphthalimide triazole **9**

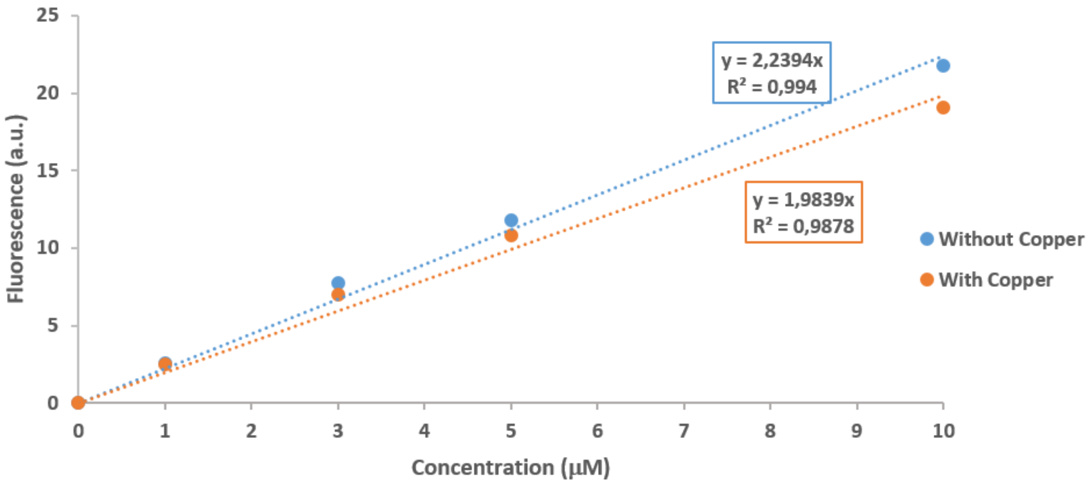

Figure S4. Calibration curve for triazole-azaphthalimide **9** determined in DMF/PBS pH 7.4 (1:3) at λ_{ex} = 320 nm, λ_{em} = 550 nm at 20 °C in the presence (orange dot) or absence of copper(I) (blue dot).

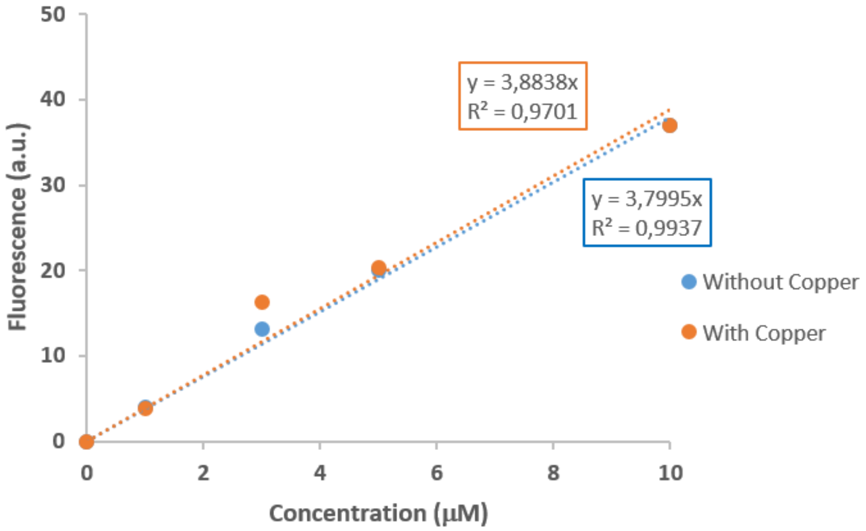

Figure S5. Calibration curve for triazole-pyridine **10** determined in DMF/PBS pH 7.4 (1:3) at λ_{ex} = 320 nm, λ_{em} = 400 nm at 20 °C in the presence (orange dot) or absence of copper(I) (blue dot).

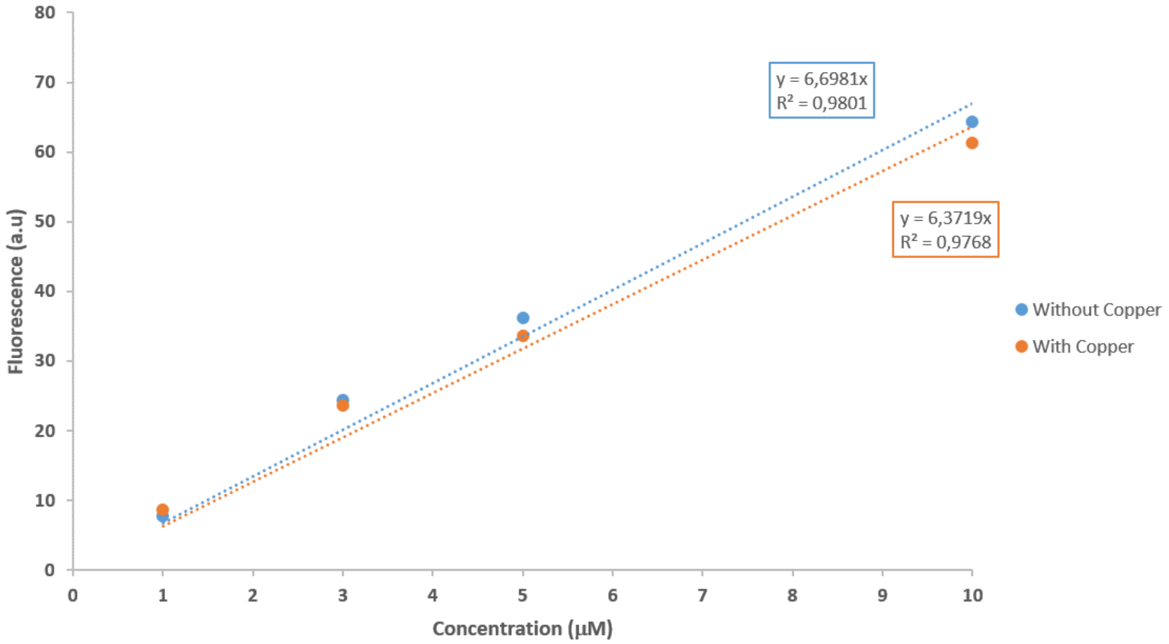

Figure S6. Calibration curve for triazole-phenyl **11** determined in DMF/PBS pH 7.4 (1:3) at λ_{ex} = 320 nm, λ_{em} = 400 nm at 20 °C in the presence (orange dot) or absence of copper(I) (blue dot).

IV. Live cells labeling:

IV.1. Preparation of stock solutions

a) Cocktail solution (with azide at 50 µM)

These 3 solutions were prepared in order to prepared the cockatail solution

1. Azide **1**: 10 mM in DMSO/milliQ water 10:90 (500 µL stock solution)
2. Copper sulfate pentahydrate: 20 mM in milliQ water (1 mL stock solution)
3. Sodium ascorbate: 0.5 M in milliQ water (1 mL stock solution)

- Cocktail solution (with azide at 50 µM): to the azide **1** stock solution (500 µL of a 10 mM solution in DMSO/milliQ water) was added the copper sulfate pentahydrate stock solution (250 µL of a 20 mM solution in milliQ water). After 30 min of incubation at 20 °C, the sodium ascorbate stock solution (250 µL of a 0.5 M solution in milliQ water) was added, and the mixture was further incubated for 15 min at 20 °C. The resulting solution was diluted 100 times with PBS 0.1 M pH 7.4. Final concentrations: azide 1 (50 µM), copper sulfate pentahydrate (50 µM, 1 eq.), sodium ascorbate (1.25 mM, 25 eq.).

b) Alkyne stock solution (50 µM)

- Alkyne 1-(4-pentyn-1-yl)-1H-pyrrole-2,5-dione **14**: a 50 µM alkyne **14** solution in PBS 0.1 M pH 7.4 (10 mL, containing 0.05% acetonitrile) was prepared from a 5 mM alkyne **14** in acetonitrile/milliQ water 5:95 (1 mL stock solution).

IV.2. Complementary confocal microscopy analysis:

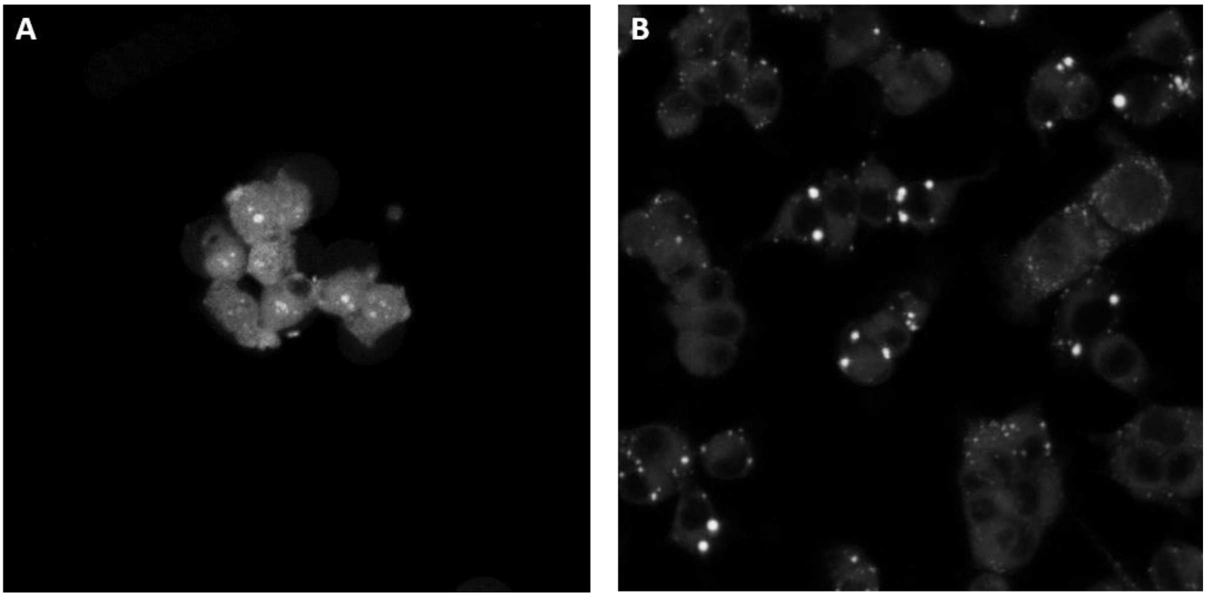

Figure S7. **A.** PC12 cells in « Cocktail+Alkyne » condition. Structure size: from 0.4 to 3.2 µm **B.** PC12 cells after BODIPY lipid probe (4,4-difluoro-1,3,5,7-tetramethyl-4-bora-3a,4a-diaza-s-indacene; 505/514 nm) staining. Lipid droplet from 0.4 to 3.9 µm. Scale bar: 10 µm.
